# Terminal sialic acids in the nanoparticle corona modulate cellular uptake

**DOI:** 10.1038/s42004-025-01677-x

**Published:** 2025-10-14

**Authors:** Marko Dobricic, Alberto Martinez-Serra, Claudia Durall, Anna Nakonechna, Jack Cheeseman, Roger Preston, James S. O’Donnell, Daniel I. R. Spencer, Teodor Aastrup, Marco P. Monopoli

**Affiliations:** 1 Department of Chemistry, Royal College of Surgeons in Ireland (RCSI), Dublin, Ireland; 2 R&D Attana AB, Sollentuna, Sweden; 3 Ludger Ltd, Culham Campus, Abingdon, Oxfordshire UK; 4 Irish Centre for Vascular Biology, School of Pharmacy and Biomolecular Sciences, Royal College of Surgeons in Ireland (RCSI), Dublin, Ireland

**Keywords:** Glycobiology, Nanotoxicology

## Abstract

Advances in engineering functional structures at the nanoscale have led to the generation of a wide range of nanoparticles (NPs) with promising therapeutic applications. However, when NPs come into contact with a biological environment, they strongly interact with the available biomolecules, such as glycoproteins. Their adsorption on the NP’s surface forms the “ biomolecular corona”. Recent findings have shown that the glycosylation of the corona affects NPs’ stability, and it is unclear whether it can engage with receptors present in the body. By dissecting the corona’s glycan composition with enzymatic approaches, we demonstrate, through differential centrifugal sedimentation and quartz crystal microbalance, that differences in the monosaccharide sialic acid content change the NP-corona interactions with isolated glycan receptors. Furthermore, flow cytometry data confirmed this behaviour in relevant cell lines. Overall, these findings highlight the role of the biomolecular corona glycosylation in NP’s interaction, suggesting advanced parameters to predict their biological fate.

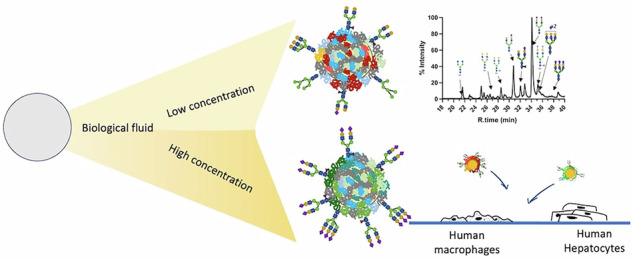

## Abstract (web summary)

Nanoparticles (NPs) hold promise for therapeutic applications, but their interactions with biological environments remain poorly understood. Here, the authors reveal that the glycosylation of the biomolecular corona, particularly sialic acid content, significantly influences NP interactions with cellular uptake processes, offering new insights into predicting NPs’ biological fate.

## Introduction

Recent advances in synthetic chemistry have brought the development of a wide range of nanomaterials that can be used in nanomedicine for drug delivery applications, diagnosis, and theragnosis^1–3^. While efforts are focused on improving their material properties and targeting abilities, concerns are growing about their potential toxicity with living systems. When nanoparticles (NP) come into contact with biological fluids such as human plasma, they are coated with a biomolecular corona layer comprising a large number of proteins and biomolecules from the biological environment^4,5^. It is well established that the biomolecular corona affects the NP’s properties in a biological milieu. The corona is composed of two layers, a loosely bound soft corona, and a more time-persistent hard corona (HC). Together, they give the nanomaterial a new biological identity that hides the originally intended surface from the body barriers and cellular receptors^6–8^. Recent studies have started correlating the nanomaterials’ circulation half-life or immunological response with the exposure of specific components and epitopes of their biomolecular corona^9–15^. With the numerous nanomedical formulations and applications, the standardised regulatory frameworks for nanomaterials in the clinic are being updated. In this context, recent guidance from the European Food Safety Authority (EFSA) acknowledges the interest in the formation and characterisation of NPs’ biomolecular corona, with recent works focusing on these methods^16–19^, and up-to-date assessments are needed to include the biomolecular corona characterisation in the regulatory framework in nanosafety and nanomedicine^20,21^.

Glycosylation is one of the proteins’ most common post-translational modifications, estimated to be present in over 50% of the human proteome^22,23^. For this reason specifically, the abundance of glycosylation in human plasma proteins has led them to be referred to as glycoproteins. Glycans are complex carbohydrates added to the protein backbone, and they play crucial roles in biological processes. Glycans are involved in a series of roles that range from correct folding and stability to cell recognition of proteins, cell-cell interaction and signalling^24–28^. Their identification by lectins, also called carbohydrate-binding proteins, can induce various responses, such as hepatic-mediated protein clearance. The exposure of the monosaccharide N-acetylneuraminic acid (referred to as sialic acid) on the glycan structure of proteins provides protection from the recognition of hepatic scavenger lectins such as macrophage galactose-type lectin (MGL) or the hepatocytic asialoglycoprotein receptor (ASGPR)^29,30^. In this clearance system, terminal sialic acid loss caused by the ageing of the proteins leads to the exposure of the underlying galactose residues, resulting in receptor recognition and immediate clearance^31,32^.

Given the importance of glycosylation in biological recognition and clearance, understanding its role in the NP biomolecular corona is vital. However, much remains unknown about how glycans within this corona layer influence NP-cell interactions^33,34^. Most biomolecular corona studies use biological fluids like blood plasma, serum, or cell culture medium, which contain a rich pool of glycosylated proteins^23^. Recent research has shown that the corona is glycosylated, that the glycans are biologically accessible for receptor binding, they can be exploited as potential biomarkers, and that they can modulate the colloidal stability of NPs^33–35^. These findings suggest that NP uptake may leverage existing physiological mechanisms for cellular uptake and clearance, indicating that glycans could play a critical role in determining the NP’s fate^36^. Emerging evidence shows that glycosylated NPs have the potential to target specific glycan receptors, and which can determine NPs fate in terms of body biodistribution^37–39^. Furthermore, glycans present on the cell membrane, specifically the glycocalyx, appear to be involved in facilitating NP internalisation^40,41^. Overall, mounting evidence is pointing to posttranslational modification in having a role on NPs’ faith.

In the current study, we aim to correlate the extrinsic physicochemical and biomolecular properties of the NPs corona with their biological response in nanomedicine studies. By exposing the NPs to 10% and 80% blood plasma, we mimicked their behaviour in different biological environments, as done in previous studies. The 10% condition was chosen to reflect standard concentrations used in in vitro experiments, while the 80% plasma was used as a more complex environment with a high-protein environment to approximate aspects of in vivo exposure^34,42^.

We showed through in-depth corona characterisation how NPs can drastically change based on the initial biological fluid concentration during the incubation step. We then assessed the accessibility of the glycans of the NP-HC complexes and their interaction profile using in situ and *in flow* methods, which showed differences in glycan epitopes. Finally, their availability for engagement was tested in relevant cell lines.

## Physicochemical and biomolecular characterisation of the NP-corona complexes

**Fig. 1:**
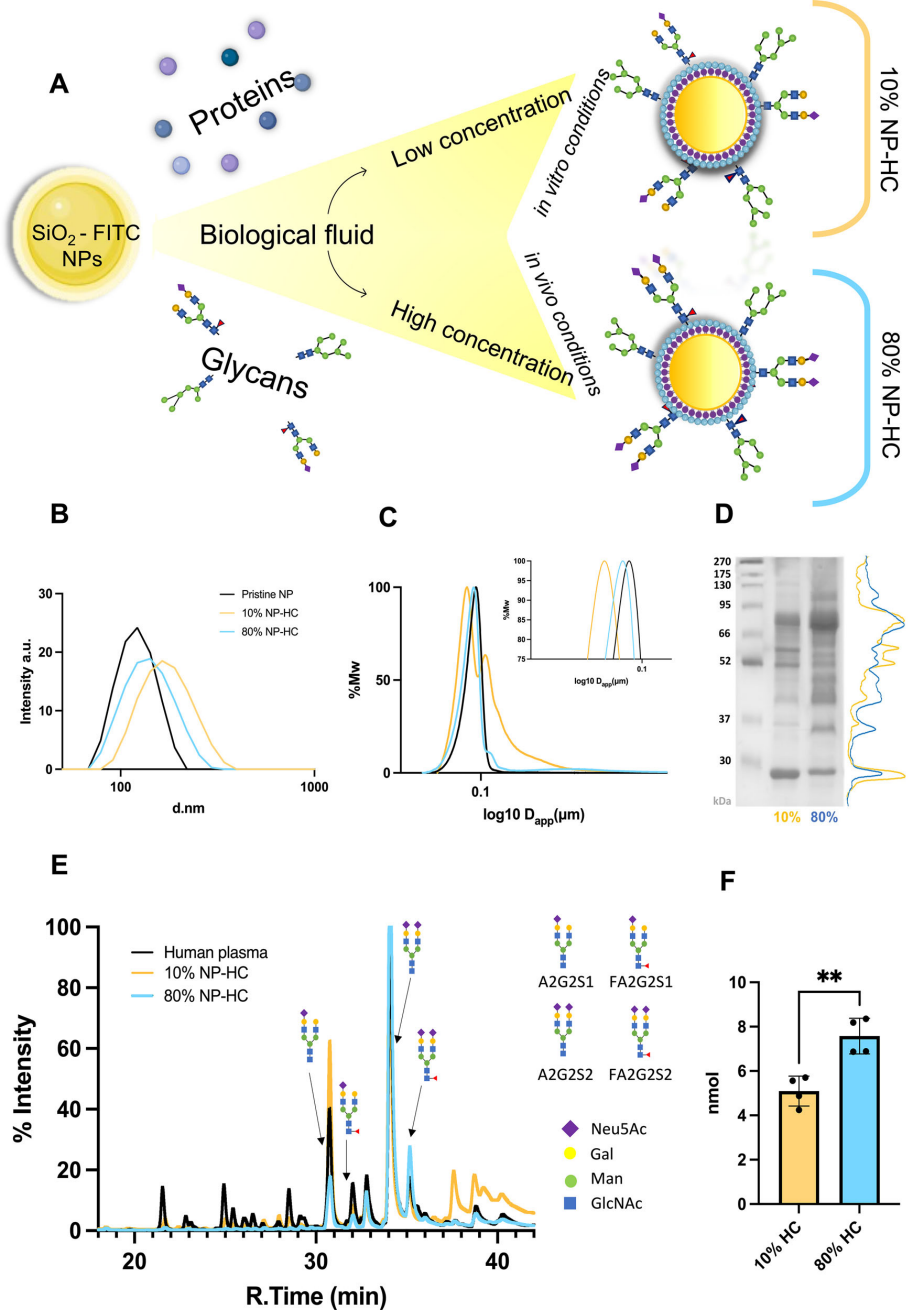
Physiochemical and biomolecular characterisation of the NP-HC complexes shows differences in the corona based on the initial concentration of human plasma. **A** Schematic showing the formation of NP–HC complexes from SiO₂–FITC nanoparticles exposed to diluted (10%) or concentrated (80%) human plasma, leading to distinct protein and glycan corona profiles. **B** Hydrodynamic diameter from DLS of the NP pristine and NP HC complexes (pristine NPs represented by a black line, 10% NP-HC complexes represented by a yellow line, and 80% NP-HC complexes represented by a green line). **C** DCS characterisation of the pristine and HC complexes, with a zoom in on the peak area. **D** SDS-PAGE of 10% and 80% NP-HC complexes and their intensity profile (10% yellow line) and 80% (blue line). **E** N-glycan profile of the NP-HC complexes after exposure to 10% (yellow line) and 80% (light blue line) plasma compared to the glycan profile of the whole plasma (black line). **F** Sialic acid analysis of 10% and 80% HC complexes. Error bars represent ± SEM; statistical significance is determined using a t-test with four biological replicates (*n* = 4), **p < 0.01.

Commercial fluorescently labelled SiO_2_ NPs of 100 nm in diameter were used in this study to isolate the biomolecular corona from human plasma at low and high protein concentrations (Fig. 1A). The were physico-chemically characterised by dynamic light scattering (DLS, Fig. 1B) and differential centrifugal sedimentation (DCS, Fig. 1C) to ensure that they were colloidally stable and within the expected size range. The NPs were then exposed to blood plasma pooled from healthy donors to allow the biomolecular corona formation for 1 h at 37 °C at low and high protein concentrations of 10% (6.11 mg/ml) and 80% (52.88 mg/ml) to mimic the in vitro and in vivo conditions. The NPs were then centrifuged at 18000 g following washing steps to remove unbound and loosely bound proteins of the soft corona, isolating the HC, which is the long-lasting and inner layer of the biomolecular corona^18^. DLS analysis showed an increase in the hydrodynamic NP size after the isolation of the NP-HC complexes (Fig. 1B, Table 1). The pristine NPs measured 104.3 ± 0.8 nm, while they measured 154.4 ± 0.7 nm and 125.7 ± 3.8 nm for the 10% and 80% conditions, respectively. These results confirmed successful biomolecular corona formation.

**Table 1:** Size distribution of SiO_2_ NPs measured by dynamic light scattering (DLS) and differential centrifugal sedimentation (DCS)

	DLS		DCS	ELS
NP-HC	*Z-average (nm)*	*PDI*	*App diameter (nm)*	*Z-potential (mV)*
Pristine	104.3 ± 0.8	0.018 ± 0.009	95.2 ± 0.8	−42.7 ± 3.1
10% NP-HC	154.4 ± 0.7	0.170 ± 0.016	88.4 ± 1.3	−12.0 ± 1.3
80% NP-HC	125.7 ± 3.8	0.105 ± 0.030	94.9 ± 1.9	−11.5 ± 0.5

Similarly, DCS analysis, a technique that measures the sedimentation time as a function of density confirmed the NP-HC complexes formation by exhibiting a peak shift in relation to pristine NPs. The peak of NP-HC complexes showed a smaller apparent diameter, 88.4 ± 1.3 nm for 10% NP-HC and 94.9 ± 1.9 nm compared to the pristine NP control, having a size of 95.2 ± 0.8 nm. This reduction NP-HC complexes compared to the pristine control, is due to changes in the NP’s total density after corona formation. Although protein adsorption increases the volume of the NPs, it lowers the average density, resulting in slower centrifugal sedimentation during DCS analysis. As a result, the recording shows a smaller size compared to bare NPs. This behaviour is consistent with findings reported in the literature (Fig. 1B, Table 1))^43^.

Interestingly, both analyses showed that 10% NP-HC complexes were greater in size compared to the 80% NP-HC ones, suggesting a more extended corona structure, as was previously seen^12,42^.

SDS-PAGE analysis also confirmed a difference in the protein composition (Figs. 1D, SI.1). NP exposure to 10% of plasma enriched bands at different molecular weights compared to 80% of plasma. Mass spectrometry analysis defined that the 10% NP-HC complexes resulted in the enriched presence of the fibrinogen alpha, beta and gamma chains, while actin cytoplasmic 1, plasminogen and serum albumin were mostly abundant on the 80% NP-HC complexes, consistent with previous findings (Table 2)^42^.

**Table 2:** E) The top 10 proteins identified in the NP-HC complexes are listed based on their label-free quantification (LFQ) values for 10% and 80% initial plasma dilution

10% NP-HC	Log_2_(LFQ)	80% NP-HC	Log_2_(LFQ)
Apolipoprotein A-I	30.4	Histidine-rich glycoprotein	32.0
Histidine-rich glycoprotein	29.4	Apolipoprotein A-I	28.8
Fibrinogen beta chain	27.6	Actin, cytoplasmic I	27.1
Fibrinogen alpha chain	27.4	Plasminogen	26.5
Apolipoprotein E	27.3	Serum Albumin	25.5
Fibrinogen gamma chain	27.2	Haemoglobin subunit beta	25.1
Coagulation factor XII	26.3	Apolipoprotein E	24.6
Kininogen-1	25.9	Ig mu chain C region	24.5
Serum amyloid A-4 protein	25.8	Talin-1	24.3
Complement C1q subunit C	25.5	Filamin-A	24.1

While changes and unicity in the protein profile were previously investigated^12,42,44^, few studies focused on the more in-depth evaluation of the glycan component^33^. To this aim, we studied the NP-HC glycan component. After isolation, the NP-HC coronas were subject to PNGase F enzyme digestion to cleave and release the N-glycan structures (Figs. SI.2 and S.3). The glycan profiles for the 10% and 80% NP-HC complexes were reproducible between pooled plasma batches and carried similar patterns to the human plasma one, differing in peak abundance (Figs. 1D,  SI.4).

Both profiles were compared to a standard glycan profile of human plasma to identify the peaks (Fig. SI.5 and Table SI.1). They showed two predominant peaks in both biomolecular coronas with an elution time of 31 and 35 min. The peaks were identified as mono-sialylated biantennary glycans (A2G2S1) and as bi-sialylated biantennary glycans (A2G2S2), respectively (Table SI.2). However, the intensity of the A2G2S1 peak of the 10% NP-HC was greater than in the 80% one, indicating a difference in the glycoprotein composition. Conversely, the intensity of the A2G2S1 glycan from the 80% NP-HC complexes was comparable to that of the human plasma control. Both coronas showed a lower enrichment of the neutral glycan structures (18–30 min glycans), which are typically abundant in immunoglobulins^45^. An increase of the A2G2S2 glycan amount at 34 mins also occurred for 80% corona compared to the 10% and plasma control (Fig. 1F). Interestingly, the glycan profile from the 10% NP-HC complexes reflected the glycan profile of fibrinogen, detected with both SDS page and mass spectrometry^23^.

While the glycan analysis confirmed a difference in the glycan types, we further quantified the specific monosaccharide sialic acid. This monosaccharide acts as a marker of self, and many immune system cells carry receptors for its recognition^46^. The monosaccharide analysis showed that the 80% NP-HC complexes had an average of 7.5 pg/ml against the 5.1 pg/ml of the 10% one (Fig. 1G). These outcomes confirmed not only the glycan structures that vary between the two corona profiles but also the abundance of the terminal monosaccharide sialic acid.

## Glycan’s biological accessibility in the corona

**Fig. 2:**
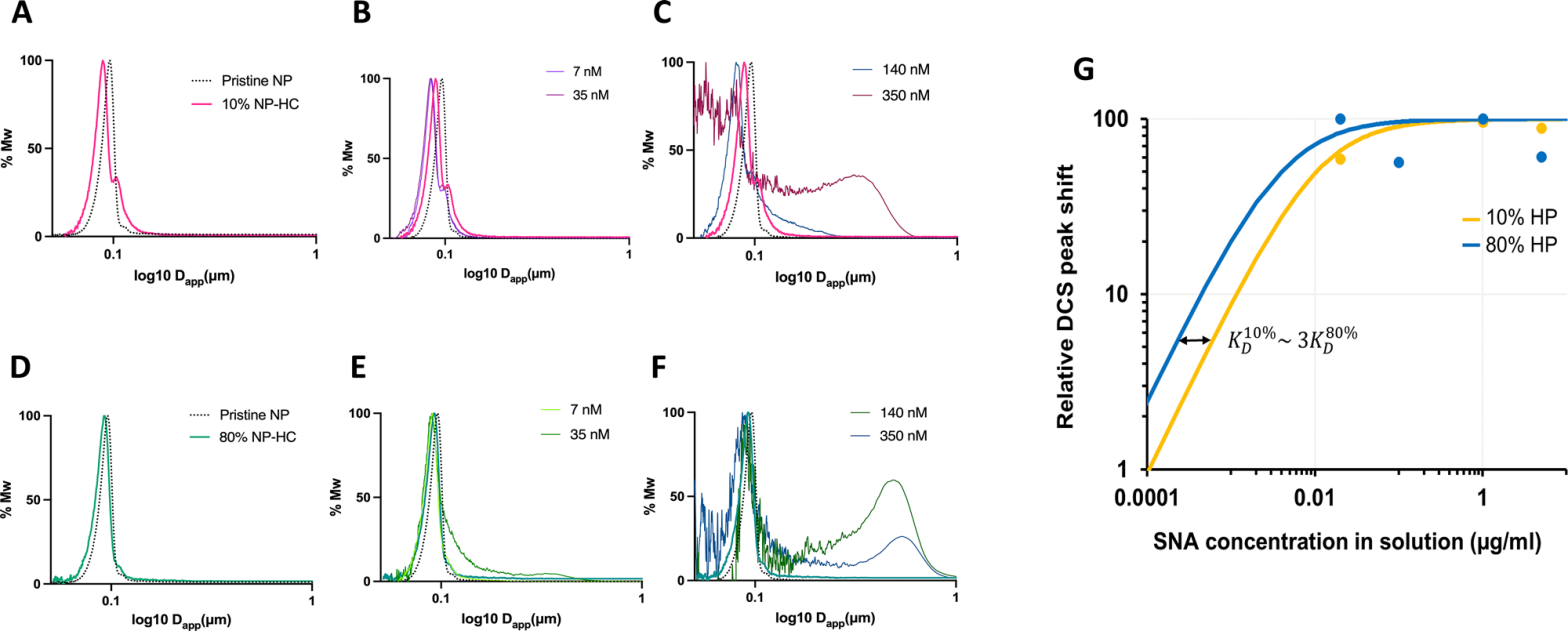
Titration experiment on 10% and 80% NP-HC complexes with Sambucus nigra agglutinin (SNA). **A**–**F** Representation of the DCS lectin binding assay using NP-HC complexes that were tested with increasing concentrations of the SNA lectin. Pristine NPs with the non-probed 10% NP-HC (**A**) and 80% NP-HC (**D**) complexes are titrated with the addition of 7 nM and 35 nM (**B** and **E**, respectively) and 140 and 350 nM (**C** and **F**, respectively). **G** Langmuir isotherms representing the different affinity of the 10% and 80% NP-HC complexes for the SNA lectin. Analysis was done on three biological replicates. *n* = 3.

To evaluate the differences in terms of glycan biological accessibility and receptor binding, we exposed the 10% and 80% NP-HC complexes to the Sambucus nigra agglutin (SNA), a lectin with affinity for sialic acid-galactose a2-6-linkage (Fig. 2). Both 10% and 80% NP-HC complexes exhibited a linear dose-response to increasing lectin concentrations. The interaction was assessed using an in situ binding assay by DCS, which showed a shift of the main peak, indicating a change in size and NP density due to lectin interactions. Initially, the apparent size of the NP-HC decreased as an indication of the formation of a two-core shell model (NP-corona-lectin complex), followed by the induced aggregation of the system. Possibly, the loss in stability due to these multiple binding sites of the lectin occurs when the lectins cover the surface of the NP-HC complexes and start cross-reacting with other NP-HC complexes, increasing the sample’s size.

Whe﻿n SNA was exposed to 10% corona, a progressive shift of the NP-corona/lectin complexes occurred towards a lower apparent size (Fig. 2A, B). However, with the exposure of a higher concentration of SNA (>140 nM) (Fig. 2C), the NP-HC stability decreased, resulting in aggregates.

A similar behaviour was observed when SNA was exposed to the 80% NP-HC complexes. However, in this case, the NP-HC complexes showed initial agglomeration levels with the presence of only 35 nM of SNA (Fig. 2D, E). Furthermore, large aggregates formed at 140 nM and 350 nM of SNA (Fig. 2F).

These findings suggest that glycans terminating in α2-6-linked sialic acid are abundant in the biomolecular corona, particularly in the 80% NP-HC complexes. Moreover, these glycans are accessible in solution and can engage with SNA. These results were also accounted for by applying a core-shell model to the peak shift to quantify the different binding affinities, providing more insights into the previous results. In particular, the dissociation constants of 10% and 80% NP-HC complexes were 1.52 mM and 0.56 mM, respectively (Fig. 2G). This indicated that SNA had three times more affinity to the sialic acid on the 80% NP-HC complexes than the 10% ones.

In the next step, we validated the biomolecular corona glycans’ ability to interact using the established *in flow* quartz crystal microbalance (QCM) biosensor^47,48^. The platform measured the binding interaction between an anti-sialic acid antibody immobilised on the chip and the sialic acid of the NP-HC complexes.

**Fig. 3:**
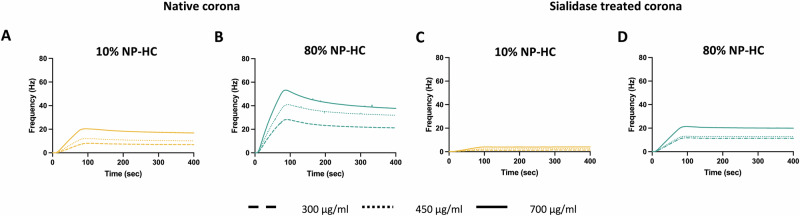
Multicycle kinetics of 10% NP-HC complexes and 80% NP-HC complexes over immobilised anti-sialic acid antibody. **A** One representative replicate (*n* = 3) of the multicycle kinetics for the 10% NP-HC (yellow lines) and **B** 80% NP-HC (green lines) complexes obtained by injecting increasing concentrations of 300 μg/mL, 450 μg/mL, and 700 μg/mL untreated **A** and **B** respectively and after sialidase treatment (**C** and **D**, respectively).

The study showed that the NP-HC complexes formed in 80% of plasma interacted more with the functionalised receptor than the 10% plasma NP-HC complexes (Fig. 3A, B). These findings confirmed that the NP-HC at 80% had a higher amount of sialic acids than the NP-HC 10% as already shown in Figs. 1E and 2G, and also indicated that these glycans were accessible for binding in solution. To validate that the interaction was due to the presence of the sialic acid on the surface of the biomolecular corona, the monosaccharides were cleaved using sialidase, which is an enzyme that cleaves the sialic acid terminal residues from a glycoprotein (Fig. SI.3). Interestingly, after the enzymatic treatment, a drop in the affinity was observed for both conditions (Fig. 3C, D). The 10% NP-HC complexes drastically decreased the interaction with the immobilised antibody on the surface, leading to weak signals for all the concentrations tested. Similarly, the 80% NP-HC complexes dropped their levels of interaction with the system, but they retained some binding capability.

## Macrophages undergo specific responses towards 10 and 80% NP-HC complexes

**Fig. 4:**
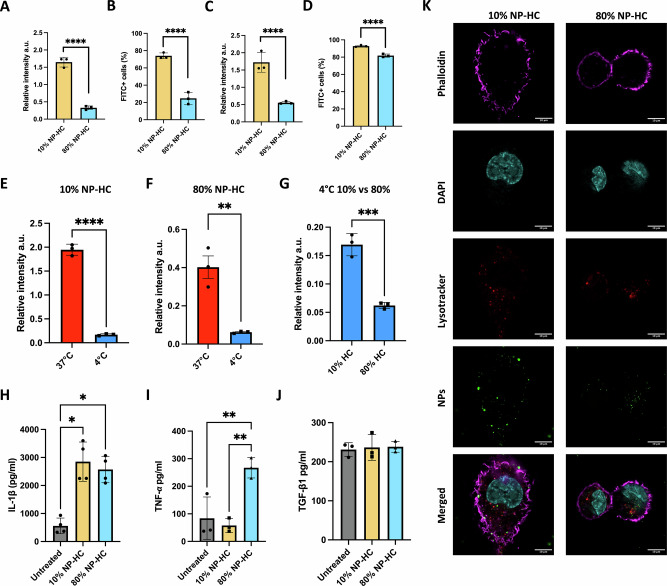
NP-HC exposure shows preferential cell uptake for 10% complexes in THP-1 macrophages. Flow cytometry analysis of 10% and 80% NP-HC complex uptake in THP-1 derived macrophages (**A**–**D**). Relative FITC intensity after 1 h and 6 h of uptake (**A**, **B**) and percentage of FITC-positive cells for 1 h and 6 h (**C**, **D**). Flow cytometry analysis of NP-HC complex uptake at 37 °C (red) and 4 °C (blue) to assess temperature-dependent membrane adhesion (**E**, **F**). Comparison of relative FITC intensities between 10% and 80% NP-HC at 4 °C after 1 h of incubation (**G**). Cytokine expression in THP-1 derived macrophages incubated with 10% and 80% NP-HC complexes at 37 °C for 5 h. IL-1β, TNF-α, and TGF-β1 levels were measured in the supernatant (**H**–**J**). Confocal microscopy images showing THP-1 macrophages after exposure to 10% NP-HC and 80% NP-HC complexes for 1 h. Phalloidin (purple), DAPI (cyan), Lysotracker (red), and NPs (green) staining are shown. Scale bars represent 10 µm (**K**). Error bars represent ± SEM; statistical significance is determined using a t-test and one-way ANOVA with Tuckey post hoc test to analyse the data of three biological replicates. *p < 0.05, **p < 0.01, ***p < 0.001, ****p < 0.0001.

Having determined that the two isolated corona complexes carried diverse physicochemical and biomolecular profiles, we next evaluated whether these differences in the biomolecular corona composition could lead to a specific biological response in immune cells. We observed that the biomolecular corona components impacted SiO_2_ NP recognition and uptake. 10% and 80% NP-HC complexes, prepared on the day of the experiments, were incubated with PMA-differentiated THP-1 macrophages in serum-free conditions for 1 h and 6 h. As the NP were fluorescently labelled, the uptake was measured with flow cytometry as the relative median cell fluorescent intensity (Fig. 4A, B, Fig. SI.7) and the number of cells positive for FITC (Fig. 4C, D). After incubation, THP-1 cells exposed to 10% NP-HC complexes exhibited significantly higher intensity values when compared to the 80% NP-HC complexes, reflecting different levels of NP uptake. This difference was maintained for both time points. In terms of the number of FITC-positive cells, we observed a different trend. After one hour, 75% of the cells exposed to the 10% NP-HC complexes showed NP internalisation, while only 35% of the cells exposed to the 80% NP-HC complexes. After 6 h, the number of cells undergoing NP internalisation increased for both conditions. In the case of cells exposed to the 10% NP-HC complexes, the peak FITC-positive cells reached 90%. Cells exposed to 80% NP-HC complexes reached 80%, with almost all the macrophages undergoing NP uptake processes (Fig. 4D). These findings showed that the NPs difference provided by the biomolecular corona impacted their recognition in a biological environment. Similar results were seen with smaller SiO_2_ NPs using human serum corona^12^.

To evaluate whether the NP-corona complexes were internalised through active uptake mechanisms and specifically recognised by macrophages, the experiments were carried out at 4 °C to deplete all energy-dependent cellular mechanisms. The relative median fluorescent intensity signals for the macrophages incubated with the NP-HC complexes showed a clear difference between experimental temperatures of 37 °C and 4 °C, decreasing signal intensity at the lower temperature (Fig. 4E, F). Interestingly, as for 37 °C, there is a difference at 4 °C in the fluorescent signal between the two conditions, suggesting specific engagement of cellular receptors (Fig. 4G).

To further assess the impact of the two biomolecular components on macrophage behaviour, we measured the cells’ cytokine release. We tested the influence of the corona complexes in inducing IL-1β and TNF-*α* production as an instance of pro-inflammatory response, while TGF-β1 as an anti-inflammatory marker. Experimental data showed higher levels of IL-1β for both coronas (Fig. 4H). In contrast, TNF-*α* increased only with the 80% NP-HC complexes (Fig. 4I). Conversely, TGF-β1 levels did not change in the presence of the NP-HC complexes (Fig. 4J). Possibly, the two corona complexes engage with different receptors, leading to distinct downstream immune responses. Together with the lower uptake of the 80% NP-HC complexes previously discussed, these results suggest a more complex series of mechanisms involved in the recognition and internalisation (Fig. 4K).

## The NP-HC complexes’ glycosylation influences cell uptake

To deepen the study of whether the biomolecular corona’s accessible glycans engage with specific glycan receptors on cell surfaces, we performed a competitive binding of NP-HC complexes and highly glycosylated proteins. We tested the uptake of NP-HC complexes in the presence of glycosylated competitors with cells known to express receptors for glycan recognition. We examined NP uptake in macrophages that express glycan-binding lectins, such as Siglecs for sialic acid, macrophage galactose lectin for galactose, and mannose-binding lectin (MBL)^46,49–51^. Additionally, we evaluated hepatocytes, which express the asialoglycoprotein receptor, specific for desialylated proteins^29,52^. We chose fetuin, which is rich in sialic acid, and asialofetuin, the asialylated proteoform, as protein competitors. Bovine serum albumin (BSA) was chosen as the non-glycosylated control^48^.

**Fig. 5:**
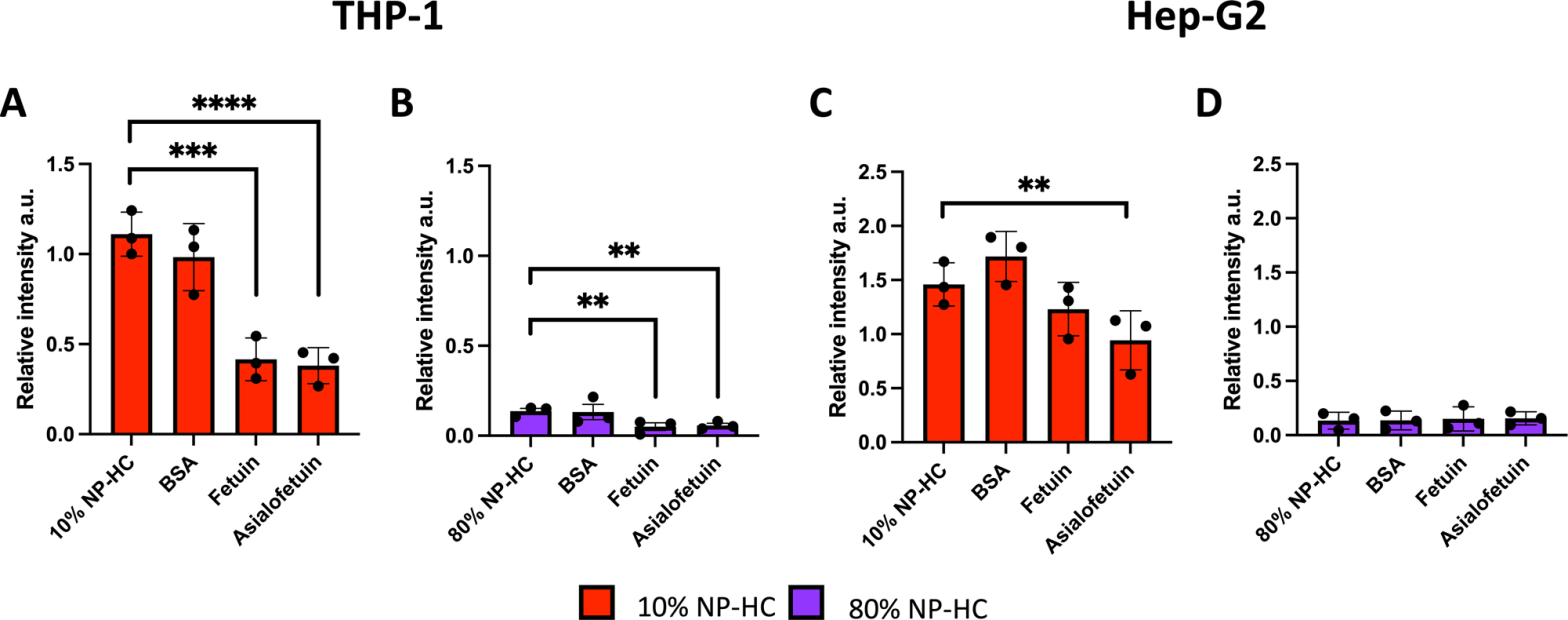
Competitive binding with glycosylated proteins reduces the NP-HC uptake in THP-1 and HepG2 cell lines. Flow cytometry analysis of competitive binding and uptake of 10% and 80% NP-HC complexes in THP-1 derived macrophages (**A**, **B**) and HepG2 hepatocytes (**C**, **D**). Cells were preincubated with 250 mM fetuin, asialofetuin, or BSA under active uptake-depleting conditions and incubated with NP-HC complexes. The relative FITC intensity is shown for 10% NP-HC (**A**, **C**) and 80% NP-HC complexes (**B**, **D**) uptake. Relative intensities are normalised to the uptake of pristine SiO_2_ NPs in full RPMI media. Data was analysed using three biological replicates error bars represent ± SEM; statistical analysis was performed using one-way ANOVA with Tukey post hoc test. **p < 0.01, ***p < 0.001, ****p < 0.0001.

As a result, in THP-1 macrophages, the uptake of the 10% NP-HC complexes was significantly affected by the presence of fetuin and asialofetuin. Both glycosylated proteins reduced the internalisation of the NPs, compared to the control. (Fig. 5A). The same result was observed for the macrophages incubated with the 80% NP-HC complexes (Fig. 5B). These outcomes show that the glycosylated proteins competed with the binding sites, leaving fewer available for interaction with the NP-HC complexes. Since immune cells, such as macrophages, express multiple glycan-epitope recognising receptors, including Siglecs (for sialic acid) and galactose lectins, it is likely that the glycan profile of the corona directly interacts with these receptors, playing a crucial role in modulating NP uptake.

On the other hand, Hep-G2 hepatocytes underwent a different trend. Only the NP-HC complexes competing with asialofetuin resulted in significantly lower internalisation (Fig. 5C, D). In fact, hepatocytes predominantly express the asialoglycoprotein receptor (ASGPR) for the recognition of desialylated proteins. Here, the presence of asialofetuin reduced NP-HC internalisation, further supporting the idea that glycans in the biomolecular corona actively engage with lectin receptors on cell surfaces. These findings reinforce that glycans of the biomolecular corona actively engage with the surface of lectin-expressing cells, indicating their role in the process.

**Fig. 6:**
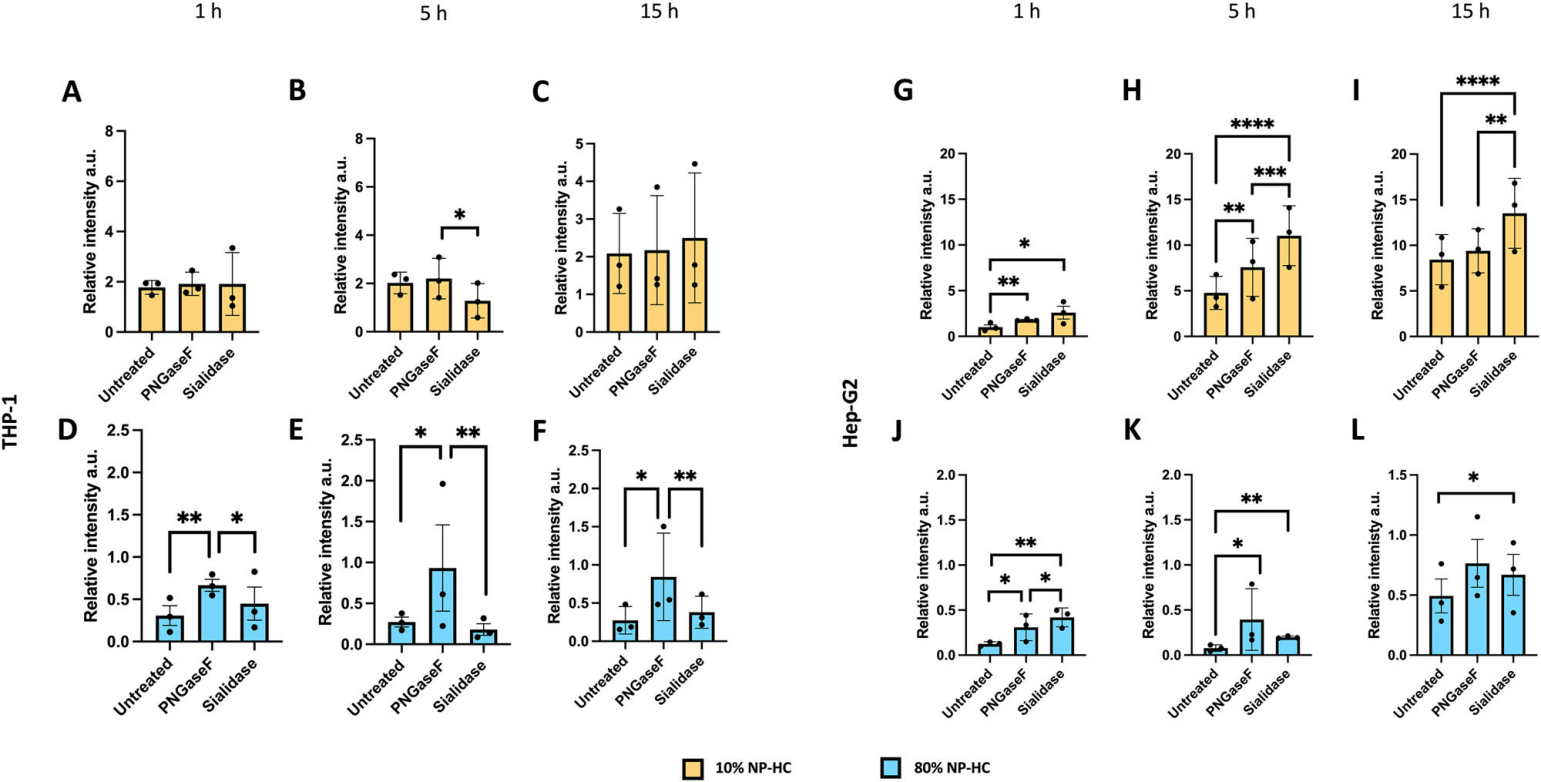
Deglycosylation of NP-HC complexes influences the cellular uptake in macrophages and hepatocytes. Deglycosylated 10% NP-HC complexes undergo preferential uptake in THP-1 macrophages (**A**–**F**) HepG2 hepatocytes (**G**–**L**) over time (1 h, 5 h, and 15 h) by flow cytometry. Each plot shows the relative intensity of the NP uptake of untreated NP-HC complexes, PNGase-F treated and sialidase-treated ones. The relative FITC intensity is shown for 10% NP-HC (**A**, **C** and **G**–**I**) and 80% NP-HC complexes (**D**–**F** and **J**–**L**) uptake. Relative intensities are normalised to the uptake of pristine SiO_2_ NPs in full RPMI media. Data represent multiple technical replicates of biological three biological replicates, error bars represent ± SEM; statistical analysis was performed using one-way ANOVA with Tukey post hoc test. *p < 0.05 **p < 0.01, ***p < 0.001, ****p < 0.0001.

Finally, we performed a series of uptake experiments after alteration of the glycosylation profile using enzymes to confirm the influence of the glycosylated component of the biomolecular corona. PNGase F, an enzyme that cleaves the N-Glycans at their base, and sialidase, which removes only the glycan’s terminal sialic acid, were employed. Following the glycan, or the monosaccharide removal, we assessed the two deglycosylated NP-HC complexes (for both 10% and 80% corona) in cell uptake using THP-1 macrophages and Hep-G2 hepatocytes at different timepoints (Fig. 6).

The results of this experiment showed that macrophages exposed to 10% NP-HC complexes carrying different glycosylation profiles were unaffected in their NP internalisation (Fig. 6A–C). Despite having no significant increase in the NP uptake, trends showed higher signals in the cases of deglycosylated NP-HC complexes. On the other hand, the removal of N-glycans with PNGase F resulted in greater uptake of the 80% corona. Conversely, in hepatocytes, sialidase-treated NP-HC complexes always showed a significant increase in uptake compared to the non-treated control. For both 10% and 80% NP-HC complexes, PNGase F-treated and sialidase-treated biomolecular coronas were more internalised, making sialidase the most effective treatment (Fig. 6G–L).

Overall, these findings are in agreement with the competitive binding assay, emphasising that uncovering the galactose residues on glycan structures significantly influences NP recognition and uptake. Therefore, variations in the biomolecular fluids used to study NPs can lead to differences in corona formation, particularly in the glycan component, which plays an active role in defining how NPs are recognised in a biological environment.

Glycosicence is, however, complex, and the presence of specific glycosylated patterns on the biomolecular corona could involve a great variety of receptors, including receptors that recognise specific danger-associated molecular patterns (DAMPs), or pathogen-associated molecular patterns (PAMPs). Depending on the cell type and the immune context, they can trigger a variety of different endocytic pathways. For instance, macrophages express lectins such as CD206 or DC-sign employed in pathogen recognition and are already being targeted for vaccine therapies^53^. A recent study showed a potent method for the identification of cellular receptors implicated in biomolecular-corona recognition and uptake^11,54,55^. Despite using different NPs, these findings highlight that the main receptors involved were MSR1 and FCR1, both directly and indirectly influenced by the protein glycosylation^55^. Further knowledge on the following endocytic mechanisms could link to how internalisation of NPs occurs. This can help the understanding of their intracellular fate, allowing to define strategies to target specific subcellular locations or to promote drug release in cells^56^.

Recognising the glycans of the biomolecular corona as a bioactive component in NP-cell interactions offers more insight into potential recognition mechanisms and subsequent material design strategies. For instance, developing materials that intrinsically adsorb highly sialylated proteins could prolong their circulation, evading clearance from immune and hepatic cells^39^. On the other hand, materials designed to adsorb less syalilated proteins could engage with macrophage and liver receptors, accumulating in the organ to target specific pathologies, or aiding for longer circulation time of parallel therapies.

## Conclusions

In summary, we demonstrated that by using the same NPs and altering the concentration of biological fluid, using 10% and 80% of plasma in PBS to mimic the in vitro and in vivo conditions, respectively, we were able to isolate two distinct NP-HC complexes differing in size, protein content, and glycosylation. These differences influenced the NPs’ interaction with cells, particularly in how they were internalised by macrophages and hepatocytes.

Our study highlights glycosylation’s functional and critical role in modulating the nanoparticle’s biomolecular corona interactions with cells. We show lower uptake trends correlated with *in-vivo like* biomolecular coronas characterised by the presence of highly sialylated glycans. We suggest that it is essential for nanomedicine research to consider the influence of biological fluid concentration and the related glycan profiles on NPs. These factors may have significant implications for the design of more effective drug delivery systems or immune-targeting therapies. Glycans can significantly modulate uptake, providing nanomaterials with a “self” layer when enriched with sialic acid or promoting targeting by specialised cells when absent. Our findings suggest new ways to explore the contribution of these post-translational modifications, highlighting the importance of integrating glycan profiles in designing and NP characterisation for therapeutic purposes. By tuning the biomolecular corona glycosylation, enhancing or reducing the terminal sialic acid content, it may be possible to influence immune recognition and biodistribution in vivo. This could lead to nanomaterials with improved circulation half-life or more organ-specific targeting.

## Materials and methods

### Materials

PBS tablets, PMA, sucrose, BSA, fetuin, and asialofetuin were purchased from Sigma Aldrich Ireland. PBS + EDTA (1 mM solution), propidium iodide, lysotracker, phalloidin, and DAPI staining solution were sourced from Invitrogen, along with trypsin for mass spectrometry sample preparation. SiO₂ nanoparticles (100 nm, PSI-G0.1) were acquired from Kisker Biotech. The CPS Disc Centrifuge DC24000 UHR and PVC calibration standard for differential centrifugal sedimentation (DCS) were obtained from Analytik Ltd, UK. The Zetasizer Nano ZS for dynamic light scattering (DLS) measurements was sourced from Malvern, UK. New England Biolabs supplied the 3x loading buffer for SDS-PAGE, and Biolegend provided the Prime-Step Prestained Broad Range Protein Ladder (6.5–270 kDa) used in gel electrophoresis. The electrophoresis system for SDS-PAGE was purchased from Bio-Rad. Imperial blue staining for SDS-PAGE was obtained from Thermo Fisher Scientific. The UltiMate3000 LC system and Orbitrap Fusion Tribrid mass spectrometer for mass spectrometry analysis were supplied by Dionex and Orbitrap, respectively, both part of Thermo Fisher Scientific. Amersham Imager 600 for gel scanning was purchased from GE Healthcare Life Sciences.

Enzymes and reagents for glycan analysis, including PNGase F enzyme, reaction buffers, LudgerTag™ DMB Sialic Acid kit, LudgerZyme PNGase F kit, and LudgerClean™ Procainamide Clean-up Plate, were purchased from Ludger LTD. R&D Systems (Abingdon, United Kingdom) provided the ELISA kits for TNF-alpha (DY-210), IL-1 beta (DY201-05), and TGF b1. Cell lines for THP-1 and HepG2 were purchased from the American Type Culture Collection (ATCC). Foetal Bovine Serum (FBS), RPMI 1640 medium, DMEM low glucose medium, and Penicillin/Streptomycin, used in cell culture, were all sourced from Gibco, part of Thermo Fisher Scientific. Sambucus nigra agglutinin for lectin binding assays was obtained from Vector Labs. The ACQUITY UPLC BEH-Glycan column for glycan UHPLC analysis was acquired from Waters. Human plasma was obtained from the Irish Blood Transfusion Service (IBTS). The use of this biological fluid for corona studies is covered by the RCSI REC 1246b.

For biosensor analysis, the Attana Cell™ 200 QCM biosensor, LNB™ carboxyl surfaces (3623-3001), and the Attana amine coupling kit (3501-3001) were sourced from Attana AB. Data analysis software included Attester™ and Evaluation™ (version 3.5.0.7), as well as TraceDrawer™ (Version 1.9.2) provided by the same supplier.

### Methods

#### Biomolecular corona preparation

Phosphate buffer is prepared by dissolving one PBS tablet in 200 mL of MilliQ water, reaching a final concentration of 0.01 M and a pH of 7.4. A volume of 6 μL from the SiO_2_ 100 nm NPs stock solution (50 mg/mL) is added to 494 mL of plasma and PBS + EDTA 1 mM solution to make a final concentration of 0.6 mg/mL of NPs. SiO_2_ NPs are incubated with the biological fluid diluted with the normalisation based on the protein concentration of 6.61 mg/ml for 10% NP-HC and 52.88 for 80% NP-HC complexes The mixture is vortexed and incubated at 37 °C under shaking conditions for 1 h. Next, the NPs-corona complexes are collected by centrifugation at 18000 *g* for 10 min. The supernatant is removed, and the pellet is resuspended in 500 μL of PBS. This process is repeated three times to obtain the NP-hard protein corona complex.

#### Dynamic light scattering (DLS)

Zetasizer Nano ZS was used for DLS measurements. The samples were prepared at an indicative concentration of 0.1 mg/mL in a volume of 500 μL. Water was used to resuspend pristine NPs, while PBS was used for the samples containing NP-protein corona complexes.

#### Differential centrifugal sedimentation (DCS)

The DCS procedure is conducted using a CPS Disc centrifuge DC24000 UHR, with a sucrose gradient ranging from 24% to 8% following the manufacturer’s protocol. The gradient is prepared by injecting 1.6 mL of 24% sucrose solution, followed by subsequent injections of the same solution, each diluted with 0.2 mL more of 8% sucrose solution, until the final injection of 1.6 mL of 8% sucrose solution. A final injection of 0.5 mL of dodecane is added to prevent gradient evaporation. For each sample, which is properly resuspended in the dispersant, 100 μg/mL is loaded onto the disc. Between each measurement, 100 μg/mL of PVC calibration standard is run to adjust the subsequent measurement as the radius increases due to the volume addition.

#### SDS-Page

After the final wash, the pellets were re-dispersed in 12 µl of mixed with 6 µl of 3x loading buffer, heated at 100 °C for 5 min and loaded for running using the Prime-Step Prestained Broad Range Protein Ladder (6.5–270 kDa) as a molecular weight marker. 12 µl of the samples were loaded in each well of 8% and 10% polyacrylamide gels prepared in the lab on the same day. Gel electrophoresis was performed with a Tris-glycine buffer on an electrophoresis system (Bio-Rad) using a voltage of 120 V, 400 mA for about 1.5 h. The gels were stained with Coomassie brilliant blue staining. Densitometry analysis was performed using FIJI.

#### Mass spectrometry sample preparation

MS samples were prepared in biological triplicates following protocols optimised in the lab^18^. The samples of the isolated coronas were run on the SDS PAGE until the blue front reached the mark at 0.5 cm below the line between the separating and stacking gels. All the area in the gels between that line and the blue front, containing the sample proteins and its vicinity, was cut. The proteins were then fixed, in-gel reduced, and alkylated before digestion with trypsin overnight at 37 °C (16 h). Subsequently, the gel pieces were subjected to peptide digestion, and the peptide digestion products were recovered and prepared with C18 tips according to the manufacturer’s protocol. The amount of peptides from each sample was quantified by Nanodrop ND-2000 prior to the MS analysis. The LC-MS/MS analysis was performed using a nanoliter flow system, an in-line connected Dionex UltiMate3000 LC system, and an Orbitrap Fusion Tribrid mass spectrometer. In brief, peptide samples were loaded onto the trapping column of PepMap100, C18, 300 μm × 5 mm, with 5 μm particle size and 100 Å pore size from TFS, for 3 min at a flow rate of 25 μL/min with 2% v/v acetonitrile, 0.1% v/v trifluoroacetic. Peptides were separated on an Acclaim PepMap 100 analytical column 75 μm × 50 cm, 3 μm bead diameter, TFS) using a binary gradient with two solvents: A: 0.1% v/v formic acid in LC-MS grade water, and B: 80% v/v acetonitrile, 0.08% v/v formic acid in LC-MS grade water; 3–50% B in 45 min, 50–90% B for 5 min and then held at 90% B for 5 min at a flow rate of 300 nL/min before returning to 3% B. MS1 spectra were acquired over m/z 380–1500 in the Orbitrap with a resolution of 120 K at 200 m/z, while the automatic gain control was set to accumulate 4 × 105 ions with a maximum injection time of 50 ms. Data-dependent tandem MS analysis was performed using a top-speed approach; cycle time was set to 3 s. Here, precursor ions were selected in the quadrupole with an isolation width of 1.6 Da. The fragmentation threshold was set at an intensity above 5000, with charge states between 2+ and 7+. Precursor ions were fragmented in the Orbitrap, 30 K resolution at 200 m/z, using HCD, with a normalised collision energy of 28%. Acquisition of MS2 spectra was done with a fixed first m/z of 110 in the ion trap. Dynamic exclusion was set at 50 s with a mass tolerance of 10 ppm. The setting of AGC was at 5 × 10^4^, and the maximum injection time was set at 300 ms.

Protein identification and quantification were performed using MaxQuant (version 2.0.3.0). The MS/MS spectra were searched using the Andromeda search engine against the forward and reversed Uniprot Homo sapiens (Human) database containing 82,518 entries (proteome ID UP000005640). Cysteine carbamidomethylation was set as a fixed modification, while N-terminal acetylation and methionine oxidation were variable modifications. Protein and peptide identifications in the present study used a 0.01 FDR threshold at both the protein and peptide levels, considering only peptides of amino-acid length seven or more. The standard target-decoy database approach was applied for other major search filtrations. Other major search parameters included a MS/MS mass tolerance of 0.02 Da, a peptide mass tolerance of 10 ppm, and tolerance for the occurrence of up to two missed cleavages. The LFQ was restricted to proteins identified with at least two unique peptides. Additionally, for a protein to be considered valid, two peptide ratios were needed. Bioinformatic analysis was performed with Perseus software (version 2.0.3.0). Missing values estimation of the LFQ intensities was carried out by imputation using a random drawing from a normal distribution.

#### N-Glycan analysis

The N-glycan release was performed in native conditions according to the protocol already described^27^. After the last wash, the NPs were resuspended in 18 μL of Milli-Q water, and both reaction buffer 10x and PNGase F enzyme were added. Incubation was performed for 1 h at 37 °C in non-shaking conditions. At the end of the incubation time, the particles were resuspended in 200 μL of PBS and centrifuged at 18000 rcf for 10 min. The supernatant was removed, and the pellet was resuspended in 100 μL of PBS.

#### Sialic acid removal

The Sialic acid release was performed in native conditions. Briefly, immediately after NP-HC complex isolation, the pellet was resuspended in 19 μL of PBS and incubated for 1 h at 37 °C. After incubation, the particles were resuspended in 200 μL of PBS and centrifuged at 18000 rcf for 10 min. The supernatant was removed, and the pellet was resuspended in 100 μL of PBS.

#### DCS-based lectin binding assay

After preparing the NP-biomolecular corona complexes and completing the final wash, the NPs were resuspended in 50 μL of PBS. Next, the tested amount of lectin is added to achieve the desired concentration in a final volume of 100 μL. The sample is then incubated at 37 °C for one hour, after which the measurement is carried out immediately using the DCS.

#### N-Glycan release

N-glycans were extracted from the protein corona using the LudgerZyme PNGase F kit. The N-glycan release was performed in native conditions. After the last wash, the NPs were resuspended in 18 μL of Milli-Q water, and both reaction buffer 10x and PNGase F enzyme were added. Incubation was performed 1 h at 37 °C in non-shaking conditions. At the end of the incubation time, the particles were resuspended in 200. This step is carried out to separate the glycans from the proteins in the solution. The samples were resuspendend in 200 μL of PBS and spun down at 18,000 rcf. The pellet was discarded, while the supernatant was collected and freeze-dried. The dried sample was incubated with a solution 1% formic acid for 50 min at room temperature in order to hydrolyse the glycosylamine and produce the labelable reducing end. Next, the glycans from the samples were separated from the proteins using a hydrophobic membrane plate and a vacuum manifold. The membrane on a 96-well plate was first activated by the addition of 100 μL of methanol in the wells and by applying a vacuum. The membranes were then cleaned and primed by adding 300 μL of water and applying a vacuum. The collection tubes were placed under the membrane, and the samples containing the glycans were added to each well and collected by applying a vacuum. The wells were washed twice with 100 μL of water, and the final collected solution was freeze-dried overnight. The samples were freeze dried ensuring that the sample dries to a small, compact mass at the very bottom of the vial. Higher temperatures than 28 °C or extremes of pH should be avoided as these conditions could result in acid-catalysed loss of sialic acids or epimerisation of the glycan reducing terminus. Once dried, 10 μL of water was added to re-dissolve glycans. The labelling was performed with the LudgerTag™ following the manufacturer’s instructions. 10 μL of a mixture of acetic acid in DMSO, procainamide dye and 2-picoline borane is added to each sample. The samples were then incubated in a heating block set at 65 °C and incubated for 1 h. To remove the free dye and unreacted chemicals, the samples were cleaned up using LudgerClean™ Procainamide Clean-up Plate per the manufacturer’s instructions.

Procainamide-labelled glycans and system standards were analysed using HILIC-UHPLC chromatography equipped with a fluorescent detector with an excitation wavelength of 310 nm and an emission wavelength of 370 nm. The ACQUITY UPLC BEH-Glycan 1.7 μm, 2.1 × 150 mm column was kept at 40 °C. Solvent A contained a 50 mM ammonium formate buffer pH 4.4, while Solvent B was acetonitrile. To prepare the samples, 75 μL of acetonitrile was mixed with 25 μL of concentrated and labelled glycans. Then, 12.5 μL of the resulting solution was injected into the column. The samples were maintained at 4 °C in the autosampler, with the oven containing the column set to 40 °C. The fluorescent unit detector parameters were adjusted to excitation 310 nm and emission 370 nm.

#### Sialic acid quantification

Release of Sialic Acid and DMB labelling of the samples was achieved using LudgerTag™ DMB Sialic Acid. After the final wash, the pellet containing the biomolecular HC complexes was resuspended in 10 μL of PBS, to which 25 μL of acetic acid 2.66 M was added. The sample was vortexed and incubated at 80 °C for 2 h in the thermoshaker. After one hour, the samples were vortexed and briefly centrifuged to ensure the correct mixing of the solution, they were briefly centrifuged and placed in the thermoshaker. At the end of the incubation time, the samples were centrifuged at 18,000 rpm to separate the NPs and the supernatant was transferred to a new tube. At this step, the samples can be labelled or stored at −20 °C for 48 h. An aliquot of 5 μL of each sample, and the sialic acid standards provided in the DMB labelling kit, were supplemented with 20 μL of a solution containing mercaptoethanol, sodium dithionite and DMB. The samples were left in incubation at 50 °C for 3 h. Every hour, they were briefly vortexed and centrifuged to ensure proper mixing of the solution. At the end of the incubation time, the samples and the standards were quenched with 475 μL and 480 μL of water, respectively. The samples were then diluted 1:10 and run on the UHPLC. DMB-labelled samples and standards were analysed using a UHPLC Shimadzu Nexera equipped with a fluorescent detector. The LudgerSep-uR2 UHPLC column was prepared, with line A containing a solution of acetonitrile:methanol:water (9:7:84) and line B consisting of acetonitrile. The samples were stored at 4 °C in the autosampler, while the oven housing the column was set to 30 °C. The fluorescence detector parameters were adjusted to an excitation wavelength of 373 nm and an emission wavelength of 448 nm. The UHPLC gradient was established per the manufacturer’s guidelines. Quantification was done by preparing a series of dilutions starting from the 1 nm standard of Neu5Ac and Neu5Gc given with the LudgerTag™ DMB-Ludger kit. Before running the actual samples, the column was conditioned with solvent A for 30 min and three water blanks were measured to make sure that the baseline is stable.

#### Cell culture

The human monocytic acute leukaemia cell line, THP-1, was cultured from passage 3 to passage 20 in RPMI 1640 medium with the supplement of 10% of Foetal Bovine Serum and 1% of Penicillin/Streptomycin. The culture is maintained in a humidified incubator at 37 °C and 5% CO_2_. To differentiate THP-1 monocytes into macrophages, 100 ng/mL PMA was added and incubated for 72 h at a concentration of 200,000 cells/mL. After differentiation, the cells were detached and transferred to a 12-well plate at a concentration of 300,000 cells/mL and left in complete growth medium overnight. Human hepatocellular carcinoma (HepG2) was cultured from passage 3 to passage 20 in DMEM low glucose medium with the supplement of 10% of Foetal Bovine Serum and 1% of Penicillin/Streptomycin. The culture is maintained in a humidified incubator at 37 °C and 5% CO_2_ and is passaged when they were 80–90% confluent. For the experiments, the cells are seeded in 12-well plates at a concentration of 200,000 cells/ml and left in a complete growth medium overnight.

#### Cellular NPs uptake by flow cytometry

The NP-corona complexes are prepared the day of the experiment and after the final wash, they are resuspended in 100 μL of PBS and 50 μL of the NPs solution is added to 2,75 mL of RPMI 1640 serum-free to reach the final concentration of 50 μg/mL. Complete growth medium is removed from the wells and 1 mL of the medium containing the NPs is added to the wells. The 12-well plates are left in the incubator at 37 °C for either 1, 5 or 15 h. After incubation, the cells are removed from the 12-well plates by gently pipetting them with PBS + EDTA 5 mM. They are then washed twice by adding PBS + 5% FBS and centrifuging them at 400 *g*. The final pellet is resuspended in 350 mL of PBS + 5% FBS. Before the flow cytometry measurement, 10 μL of 1 mg/mL propidium iodide solution is added to the tube to visualise the dead cells. For each sample, triplicates were made, and each experiment was performed three times.

BSA, fetuin, and asialofetuin for the protein competitive binding experiments are dispersed in serum-free RPMI 1640. The enriched media was filtered using 0.21 μm sterile filters and was added to the cells for 1 h at 4 °C. The medium was then removed, and the cells were incubated with serum-free media containing NPs and the plate was left for 1 h at 37 °C. The NP uptake was then assessed using flow cytometry.

#### Confocal microscopy

On the first day, coverslips were sprayed with 70% ethanol and placed under a laminar flow hood to dry. Once dry, the coverslips were placed at the bottom of a 6-well plate, and two edges were sealed with nail varnish. After letting the varnish dry, the wells were washed twice with 1x PBS. A 0.5% gelatine solution was then added to the wells, and the 6-well plate was incubated at 37 °C for 30 min. Following incubation, any leftover solution was removed, and the wells were washed 2–3 times with 1x PBS. Cells were then seeded at the desired concentration and left to adhere overnight.

On the second day, cells were incubated with NPs. After incubation, the NP solution was removed, and the well surface was washed three times with PBS. The cells were then incubated with a Lysotracker solution diluted 1:500 or 1:1000 for 30 min, followed by fixation using 4% formaldehyde for 15 min. The wells were then washed three times with 1x PBS. To block non-specific binding, 1% BSA was added for 15 min, followed by three washes with 1x PBS.

Coverslips were detached using tweezers and placed on hydrophobic weighing boards. Next, 200 µL of phalloidin (5 µL of stock phalloidin + 195 µL of PBS) was added to each coverslip, and samples were incubated in the dark for 30 min. After incubation, the coverslips were washed three times with 1x PBS. A 300 nM DAPI staining solution was added to the samples, and they were incubated for 1–5 min (2.5 min recommended). Following this, the coverslips were washed three times with 1x PBS.

A PAP pen was used to draw a circle on a microscope slide, and 20–25 µL of mounting media was added to the centre. The glass side of the coverslip was dried, and any residual PBS was removed using a paper towel, being careful not to touch the cells. Finally, the coverslip was placed cell-side-down onto the mounting media on the microscope slide, and the edges were sealed with nail varnish.

#### Cytokine assay

Human TNF-alpha and Human IL-1 beta DuoSet® ELISA assays were performed according to manufacturers’ instructions. A standard curve was prepared by serial dilution of purified recombinant protein supplied with the kit (starting from 1000 pg/mL for TNF-alpha 250 pg/mL for IL-1 beta). To avoid the presence of the NPs in the solution, before the beginning of the experiment, the previously collected cell supernatants were centrifuged at 18000 *g* for 10 min, and the pellet was discarded. The resulting optical density was determined using the CLARIOStar automatic plate reader at 450 nm with a wavelength correction reading at 570 nm. The concentrations of the unknowns were calculated from the standard curve.

#### Statistical analysis

Statistical analysis was carried out using Graph-Pad Prism 9.2. Where two treatments were compared, an unpaired, two-tailed *t*-test was used. Where more than one treatment was compared, a one-way ANOVA with a Tukey post-hoc test was carried out. All experiments were performed in triplicate and were compromised of multiple technical replicates. Results are expressed as mean ± standard error of the mean.

#### QCM biosensor development

The Attana Cell™ 200 QCM biosensor setup involves two primary stages: immobilisation of the ligand onto the chip surface and the kinetic interaction between the ligand and the injected analyte during the experiment. SNA immobilisation (50 μg/mL) was carried out on LNB™ carboxyl surfaces (3623-3001) following the Attana amine coupling kit protocol Immobilisation of PNA (50 μg/mL) on LNB™ carboxyl surfaces was performed according to the Attana amine coupling kit. Immobilisation of anti-sialic acid antibody was performed on LNB™ carboxyl surfaces according to the Attana amine coupling kit. Kinetic experiments were conducted at a flow rate of 0.1 mL/min and a temperature of 22 °C. All tests were performed with a continuous flow of PBS. A PBS blank injection preceded each analyte injection. The blank injection was then subtracted from the following analyte injection to correct for baseline drift. Individual analytes were injected for 90 s over the cell surfaces. Four two-fold dilutions of each analyte were introduced over cell surfaces for kinetic experiments. Surface regeneration was executed through a 30-second injection of 10 mM glycine (pH 1). Repeated injections of the same analyte concentration produced consistent binding curves, indicating that regeneration did not affect the binding capacity of the surface. The sensor surface resonance frequency change (ΔF) during binding experiments was recorded using the Attester™ software, and the data were analysed with Evaluation™ and TraceDrawer™ software. The analysis used 1:1 or 1:2 binding models to compute the kinetic parameters, including rate constants (k_on_, k_off_), dissociation equilibrium constant (K_D_), and the maximum binding capacity (B_max_). Simulation curves were generated with the Evaluation software.

### Reporting Summary

Further information on research design is available in the Nature Portfolio Reporting Summary linked to this article.

## Supplementary information

Supplemental Information

Nr Reporting summary

## Data Availability

The data supporting this study are provided in the main article and in the supplementary information. Raw data are available at: 10.5281/zenodo.16795725.

## References

[1] Pelaz, B. et al. Diverse applications of nanomedicine. *ACS Nano* **11**, 2313–2381 (2017). 28290206 10.1021/acsnano.6b06040 PMC5371978

[2] Walkey, C. D., Olsen, J. B., Guo, H., Emili, A. & Chan, W. C. W. Nanoparticle size and surface chemistry determine serum protein adsorption and macrophage uptake. *J. Am. Chem. Soc.* **134**, 2139–2147 (2012). 22191645 10.1021/ja2084338

[3] Mitchell, M. J. et al. Engineering precision nanoparticles for drug delivery. *Nat. Rev. Drug Discov.*10.1038/s41573-020-0090-8 (2020). 33277608 10.1038/s41573-020-0090-8 PMC7717100

[4] Monopoli, M. P., Åberg, C., Salvati, A. & Dawson, K. A. Biomolecular coronas provide the biological identity of nanosized materials. *Nat. Nanotechnol.* **7**, 779–786 (2012). 23212421 10.1038/nnano.2012.207

[5] Mahmoudi, M., Landry, M. P., Moore, A. & Coreas, R. The protein corona from nanomedicine to environmental science. *Nat. Rev. Mater.* **8**, 422–438 (2023). 10.1038/s41578-023-00552-2 PMC10037407 37361608

[6] Nel, A. E. et al. Understanding biophysicochemical interactions at the nano-bio interface. *Nat. Mater.* **8**, 543–557 (2009). 19525947 10.1038/nmat2442

[7] Casals, E., Vitali, M. & Puntes, V. The nanoparticle-Protein Corona untold history (1907–2007). *Nano Today* **58**, 102435 (2024).

[8] Dawson, K. A. & Yan, Y. Current understanding of biological identity at the nanoscale and future prospects. *Nat. Nanotechnol.* **16**, 229–242 (2021). 33597736 10.1038/s41565-021-00860-0

[9] Saha, K. et al. Regulation of macrophage recognition through the interplay of nanoparticle surface functionality and protein Corona. *ACS Nano* **10**, 4421–4430 (2016). 27040442 10.1021/acsnano.6b00053 PMC5696791

[10] Chen, F. et al. Complement proteins bind to nanoparticle protein corona and undergo dynamic exchange in vivo. *Nat. Nanotechnol.* **12**, 387–393 (2017). 27992410 10.1038/nnano.2016.269 PMC5617637

[11] Ngo, W. et al. Identifying cell receptors for the nanoparticle protein corona using genome screens. *Nat. Chem. Biol.* **18**, 1023–1031 (2022). 35953550 10.1038/s41589-022-01093-5

[12] Francia, V. et al. Corona composition can affect the mechanisms cells use to internalize nanoparticles. *ACS Nano* **13**, 11107–11121 (2019). 31525954 10.1021/acsnano.9b03824 PMC6812477

[13] Ambrosone, A. et al. Impact of Amorphous SiO_2_ Nanoparticles on a Living Organism: Morphological, Behavioral, and Molecular Biology Implications. *Front. Bioeng. Biotechnol.* **2**, 10.3389/fbioe.2014.00037 (2014). 10.3389/fbioe.2014.00037 PMC4179610 25325055

[14] Barbero, F. et al. Formation of the Protein Corona: The interface between nanoparticles and the immune system. *Semin Immunol.* **34**, 52–60 (2017). 29066063 10.1016/j.smim.2017.10.001

[15] Skuland, T. et al. Pro-inflammatory effects of crystalline- and nano-sized non-crystalline silica particles in a 3D alveolar model. *Part Fibre Toxicol.* **17**, 13 (2020). 32316988 10.1186/s12989-020-00345-3 PMC7175518

[16] Committee, E. S. et al. Guidance on risk assessment of nanomaterials to be applied in the food and feed chain: human and animal health. *EFSA J.* **19**, e06768 (2021). 34377190 10.2903/j.efsa.2021.6768 PMC8331059

[17] Francia, V. et al. A magnetic separation method for isolating and characterizing the biomolecular corona of lipid nanoparticles. *Proc. Natl. Acad. Sci.* **121**, e2307803120 (2024). 38437542 10.1073/pnas.2307803120 PMC10945860

[18] Soliman, M. G. et al. Protocols for isolation and characterization of nanoparticle biomolecular corona complexes. *Front. Toxicol.* **6**, 10.3389/ftox.2024.1393330 (2024). 10.3389/ftox.2024.1393330 PMC11301017 39109300

[19] Usmani, S. M. et al. Review of new approach methodologies for application in risk assessment of nanoparticles in the food and feed sector: status and challenges. *EFSA Support. Publ.* **21**, 8826E (2024).

[20] Centre, E. C. J. R. et al. *Anticipation of Regulatory Needs for Nanotechnology-Enabled Health Products – The REFINE White Paper*. (Publications Office, 2019). 10.2760/596822.

[21] Halamoda-Kenzaoui, B. et al. Future perspectives for advancing regulatory science of nanotechnology-enabled health products. *Drug Deliv. Transl. Res* **12**, 2145–2156 (2022). 35691982 10.1007/s13346-022-01165-y PMC9360093

[22] Helenius, A. & Aebi, M. Intracellular functions of N-linked glycans. *Science (1979)* **291**, 2364–2369 (2001). 10.1126/science.291.5512.2364 11269317

[23] Clerc, F. et al. Human plasma protein N-glycosylation. *Glycoconj. J.* **33**, 309–343 (2016). 10.1007/s10719-015-9626-2 PMC4891372 26555091

[24] Helenius, A. & Aebi, M. Roles of N-linked glycans in the endoplasmic reticulum. *Annu. Rev. Biochem* **73**, 1019–1049 (2004). 15189166 10.1146/annurev.biochem.73.011303.073752

[25] Collins, B. E. & Paulson, J. C. Cell surface biology mediated by low-affinity multivalent protein-glycan interactions. *Curr. Opin. Chem. Biol.* **8**, 617–625 (2004). 15556405 10.1016/j.cbpa.2004.10.004

[26] Takahashi, M., Hasegawa, Y., Gao, C., Kuroki, Y. & Taniguchi, N. N-glycans of growth factor receptors: their role in receptor function and disease implications. *Clin. Sci.* **130**, 1781–1792 (2016). 10.1042/CS20160273 27612953

[27] Fiedler, K. & Simons, K. The role of n-glycans in the secretory pathway. *Cell* **81**, 309–312 (1995). 7736583 10.1016/0092-8674(95)90380-1

[28] Mariño, K., Bones, J., Kattla, J. J. & Rudd, P. M. A systematic approach to protein glycosylation analysis: a path through the maze. *Nat. Chem. Biol.* **6**, 713–723 (2010). 20852609 10.1038/nchembio.437

[29] Rice, K. G. & Lee, Y. C. Modification of triantennary glycopeptide into probes for the asialoglycoprotein receptor of hepatocytes. *J. Biol. Chem.* **265**, 18423–18428 (1990). 2211710

[30] Mortezai, N. et al. Tumor-associated Neu5Ac-Tn and Neu5Gc-Tn antigens bind to C-type lectin CLEC10A (CD301, MGL). **23**, 844–852 (2013). 10.1093/glycob/cwt021 23507963

[31] Ward, S., O’Sullivan, J. M. & O’Donnell, J. S. von Willebrand factor sialylation—A critical regulator of biological function. *J. Thromb. Haemost.* **17**, 1018–1029 (2019). 31055873 10.1111/jth.14471

[32] O’Sullivan, J. M., Ward, S., Lavin, M. & O’Donnell, J. S. von Willebrand factor clearance – biological mechanisms and clinical significance. *Br. J. Haematol.* **183**, 185–195 (2018). 30378120 10.1111/bjh.15565

[33] Wan, S. et al. The “ Sweet ” side of the Protein Corona: Effects of Glycosylation on nanoparticle À cell interactions. 2157–2166 10.1021/nn506060q (2015). 10.1021/nn506060q 25599105

[34] Clemente, E. et al. Probing the glycans accessibility in the nanoparticle biomolecular corona. *J. Colloid Interface Sci.* **613**, 563–574 (2022). 35066229 10.1016/j.jcis.2021.11.140

[35] Trinh, D. N. et al. Nanoparticle biomolecular corona-based enrichment of plasma glycoproteins for N-glycan profiling and application in biomarker discovery. *ACS Nano* **16**, 5463–5475 (2022). 35341249 10.1021/acsnano.1c09564 PMC9047655

[36] Fadeel, B. Hide and seek: nanomaterial interactions with the immune system. *Front Immunol.* **10**, 1–10 (2019). 30774634 10.3389/fimmu.2019.00133 PMC6367956

[37] Ferreira, A. et al. Tuning of Ultrasmall gold nanoparticles surface properties affect their biological fate. *Part. Part. Syst. Charact.*10.1002/ppsc.202300168 (2024).

[38] Soliman, M. G. et al. Decoration of gold nanoparticles with glycopeptides leads to a lower cellular uptake and liver retention. *Nanoscale Adv.*10.1039/D5NA00464K (2025). 10.1039/d5na00464k PMC12341466 40808819

[39] Ahmad, A. et al. Polymer-tethered glycosylated gold nanoparticles recruit sialylated glycoproteins into their protein corona, leading to off-target lectin binding. *Nanoscale* **14**, 13261–13273 (2022). 36053227 10.1039/d2nr01818g PMC9494357

[40] Olivieri Jr, P. H. et al. Glycocalyx interactions modulate the cellular uptake of Albumin-coated nanoparticles. *ACS Appl. Bio. Mater.* **7**, 7365–7377 (2024). 39470630 10.1021/acsabm.4c01012 PMC11577421

[41] Bussin, B., MacDuff, M. G. G., Ngo, W. & Chan, W. C. W. Cellular Glycocalyx affects nanoparticle access to cell membranes and uptake. *Adv. Mater.*10.1002/adma.202503004 (2025). 40269604 10.1002/adma.202503004

[42] Monopoli, M. P. et al. Physical-chemical aspects of protein corona: Relevance to in vitro and in vivo biological impacts of nanoparticles. *J. Am. Chem. Soc.* **133**, 2525–2534 (2011). 21288025 10.1021/ja107583h

[43] Perez-Potti, A. et al. In depth characterisation of the biomolecular coronas of polymer coated inorganic nanoparticles with differential centrifugal sedimentation. *Sci. Rep.* **11**, 6443 (2021). 33742032 10.1038/s41598-021-84029-8 PMC7979877

[44] Walczyk, D. et al. Physical−chemical aspects of Protein Corona: Relevance to in vitro and in vivo biological impacts of nanoparticles. *J. Am. Chem. Soc.* **133**, 2525–2534 (2011). 21288025 10.1021/ja107583h

[45] Cobb, B. A. The history of IgG glycosylation and where we are now. *Glycobiology* **30**, 202–213 (2020). 31504525 10.1093/glycob/cwz065 PMC7109348

[46] Varki, A. Letter to the Glyco-Forum: Since there are PAMPs and DAMPs, there must be SAMPs? Glycan ‘self-associated molecular patterns’ dampen innate immunity, but pathogens can mimic them. *Glycobiology* **21**, 1121–1124 (2011). 21932452 10.1093/glycob/cwr087 PMC3150115

[47] Gianneli, M. et al. Label-free in-flow detection of receptor recognition motifs on the biomolecular corona of nanoparticles. *Nanoscale* **10**, 5474–5481 (2018). 29511756 10.1039/c7nr07887k

[48] Cummings, R. D. et al. Glycan-recognizing probes as tools. 10.1101/glycobiology.4e.48 (2022). 35536983

[49] Läubli, H. & Varki, A. Sialic acid–binding immunoglobulin-like lectins (SIGLECS) detect self-associated molecular patterns to regulate immune responses. *Cell. Mol. Life Sci.* **77**, 593–605 (2020). 31485715 10.1007/s00018-019-03288-x PMC7942692

[50] Ward, S. E. et al. A novel role for the macrophage galactose-type lectin receptor in mediating von Willebrand Factor clearance. 10.1182/blood-2017-06-787853 (2018). 10.1182/blood-2017-06-787853 29282218

[51] Ward, S. E. et al. Macrophage Galactose Lectin contributes to the regulation of FVIII (Factor VIII) Clearance in Mice—Brief Report. *Arterioscler. Thromb. Vasc. Biol.* **43**, 540–546 (2023). 36727518 10.1161/ATVBAHA.122.317807 PMC10026961

[52] Ashwell, G. & Morell, A. G. The role of surface carbohydrates in the hepatic recognition and transport of circulating glycoproteins. *Adv. Enzymol. Relat. Areas Mol. Biol.* 99–128 10.1002/9780470122860.ch3. (1974). 10.1002/9780470122860.ch3 4609051

[53] Nahar, U. J., Toth, I. & Skwarczynski, M. Mannose in vaccine delivery. *J. Control. Release* **351**, 284–300 (2022). 36150579 10.1016/j.jconrel.2022.09.038

[54] Montizaan, D. et al. Genome-wide forward genetic screening to identify receptors and proteins mediating nanoparticle uptake and intracellular processing. *Nat. Nanotechnol.* **19**, 1022–1031 (2024). 38504023 10.1038/s41565-024-01629-x

[55] Bussin, B. et al. Discovering nanoparticle corona ligands for liver macrophage capture. *Nat. Nanotechnol.*10.1038/s41565-025-01903-6 (2025). 40374797 10.1038/s41565-025-01903-6

[56] Francia, V., Montizaan, D. & Salvati, A. Interactions at the cell membrane and pathways of internalization of nano-sized materials for nanomedicine. *Beilstein J. Nanotechnol.* **11**, 338–353 (2020). 32117671 10.3762/bjnano.11.25 PMC7034226

