# Supplementary material for: Terminal sialic acids in the nanoparticle corona modulate cellular uptake
Source: Commun Chem. 2025 Oct 14;8:308. doi: 10.1038/s42004-025-01677-x (PMC12521558; doi:10.1038/s42004-025-01677-x)
Supplement: Supplementary file 1 — Supplemental Information [file 42004_2025_1677_MOESM1_ESM.pdf]

Supporting Information

Terminal Sialic Acids in the Nanoparticle Corona Modulate Cellular Uptake

Marko Dobricic[1], Alberto Martinez-Serra[1], Claudia Durall[2], Anna Nakonechna[1], Jack Cheeseman[3], Roger Preston[4], James S O'Donnell[4], Daniel IR Spencer[3], Teodor Aastrup[2], Marco P Monopoli[1]

[1] Department of Chemistry, Royal College of Surgeons in Ireland (RCSI), Dublin 2, Ireland
[2] R&D Attana AB, Sollentuna, Sweden
[3] Ludger Ltd, Culham Campus, Abingdon, Oxfordshire, United Kingdom, OX14 3EB
[4] Irish Centre for Vascular Biology, School of Pharmacy and Biomolecular Sciences, Royal College of Surgeons in Ireland (RCSI), Dublin 2, Ireland

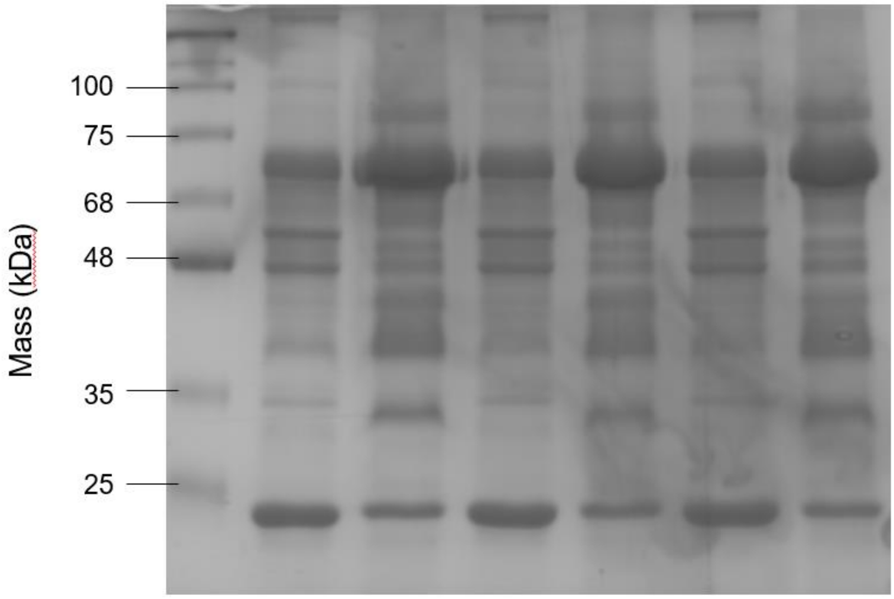

Figure SI.1: *Unprocessed SDS Gel electrophoresis image of the NP-HC in 10 and 80% of human plasma. The following samples were loaded from left to right: Lane 1: Molecular marker; 2,4,6:* SiO_2 *– NP HC 10% human plasma;3,5,7:* SiO_2 *– NPHC 80% human plasma*

A

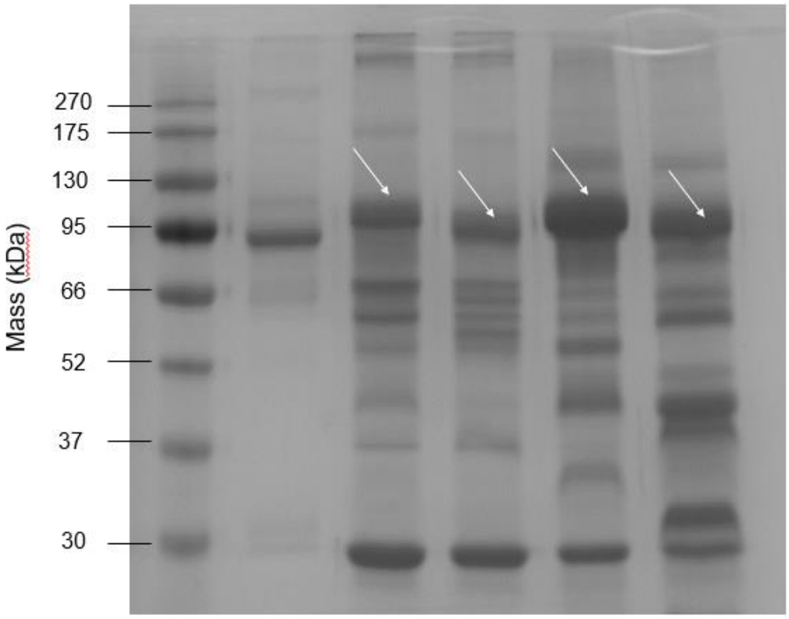

Figure SI.2: *(A) PNGase F enzymatic efficiency visualised by gel electrophoresis with highlight of bands dropping in molecular weight. The following samples were loaded from left to right: Lane 1: Molecular marker, 2: Full plasma, 3: 10% NP HC, 4: 10% NP HC PNGase F treated, 5: 80% NP HC, 6: 80% NP HC PNGase F treated.*

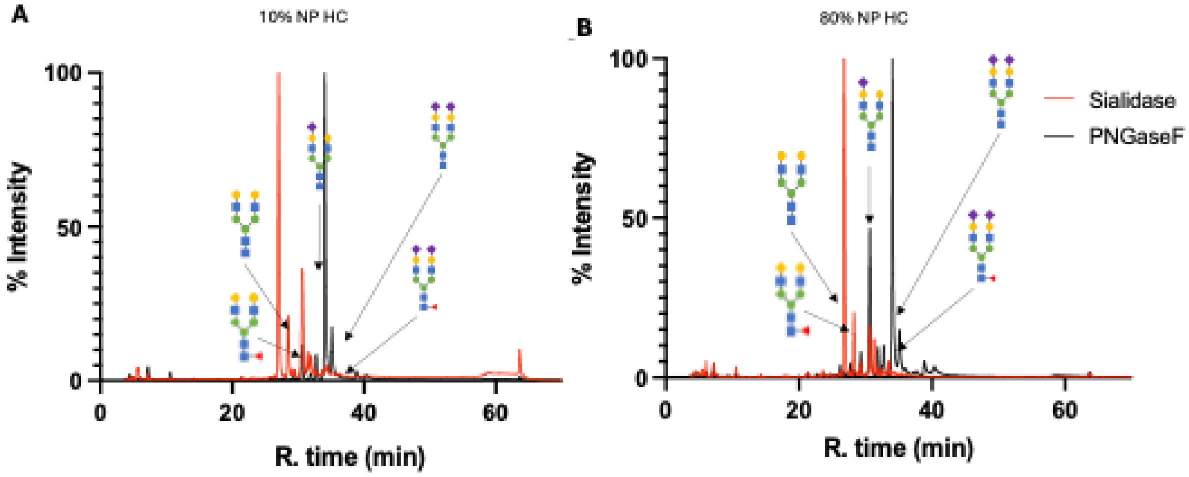

Figure SI.3: *(B) Glycan analysis pre (black) and post sialidase treatment (red) for 10% (A) and 80% NP HC (B). The highlighted glycans are A2G2S1 and A2G2S2, alongside the fucosilated form and desyalilated one.*

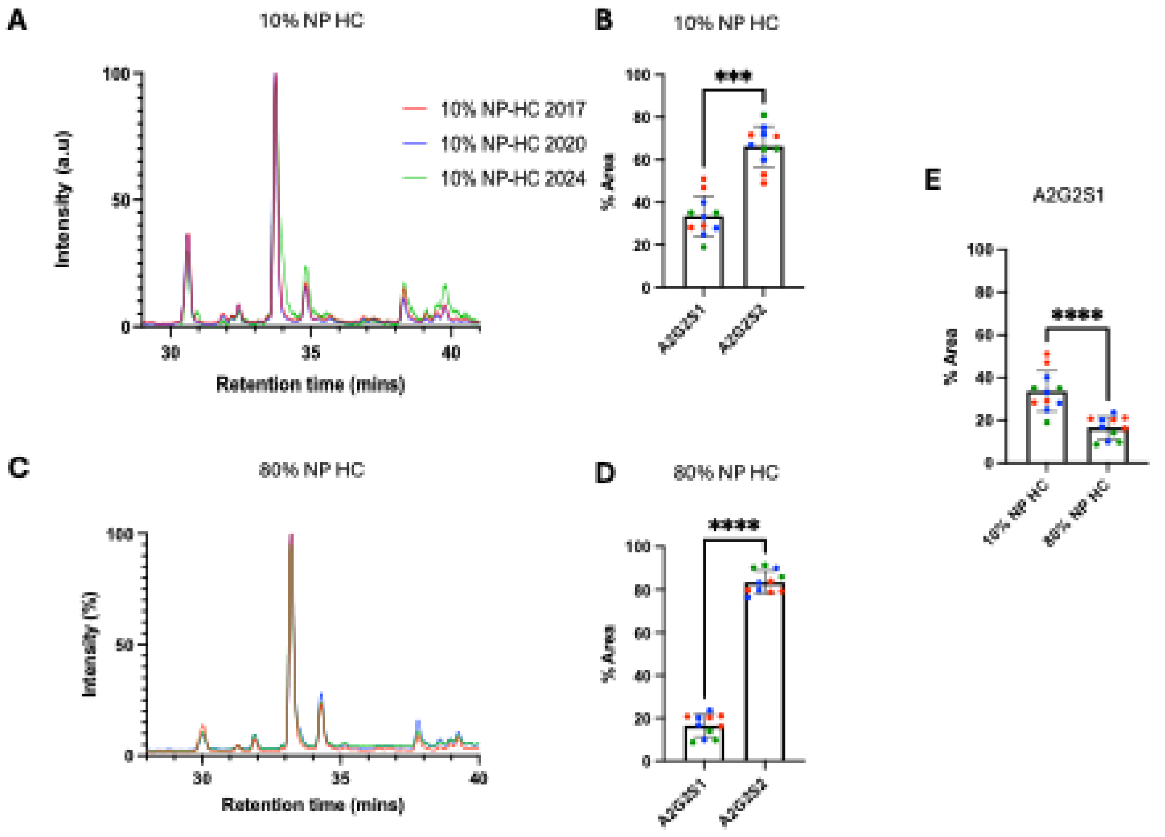

Figure SI.4: *Glycan release across plasma batches of pooled plasma. Overlap of three glycan profiles obtained with the use of three different PNGaseF enzymes on 10% NP HC samples over three different batches of pooled plasma from eight donors (A). Reproducibility was evaluated by integrating the relative area of the main peaks A2G2S1 and A2G2S2. The same analysis was done for 80% NP HC samples (C and D, respectively). Comparison between the A2G2S1 peak relative area from 10% and 80% NP HC samples shows the prevalence of the peak in 10% NP HC.*

Pooled plasma batches were color-coded: Batch 1 (blue), Batch 2 (green), and Batch 3 (red). Chromatograms are shown from retention time 25 to 45 minutes. Quantitative analysis of A2G2S1 and A2G2S2 glycan peaks (F and G), and of the A2G2S1 glycan (H) over the two conditions. Statistical analysis was performed using paired t-tests on

technical replicates (Batch 1 n= 4, Batch 2 n= 4, Batch 3 n= 3) per pooled plasma batch. ***p < 0.001, ***p < 0.0001.

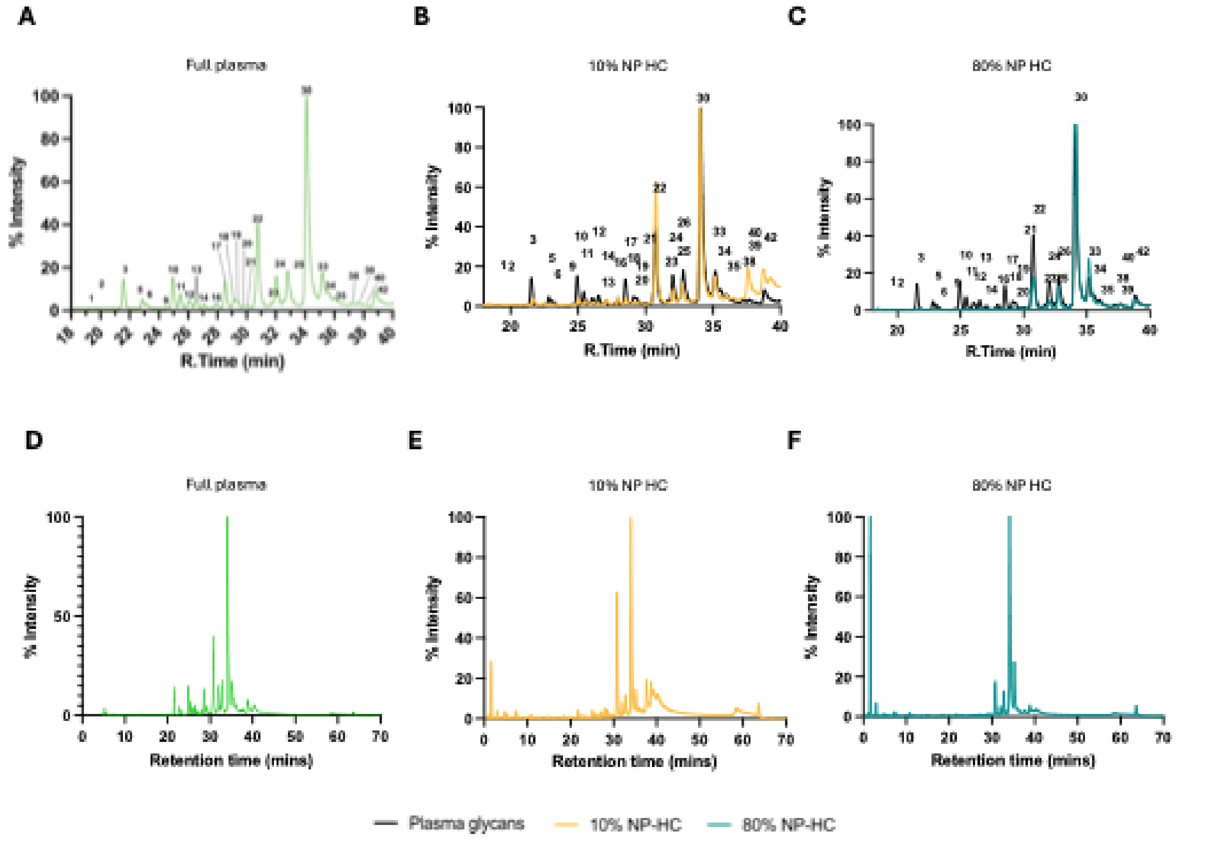

Figure SI.5: *Glycan analysis peak definition (A) Glycan peaks were assigned using standard libraries provided by Ludger Ltd. Overlay of pooled plasma glycan profiles enabled identification of corresponding peaks in 10% NP-HC (B) and 80% NP-HC (C) corona fractions. Full glycan profiles are shown for whole plasma (D), 10% NP-HC (E), and 80% NP-HC (F).*

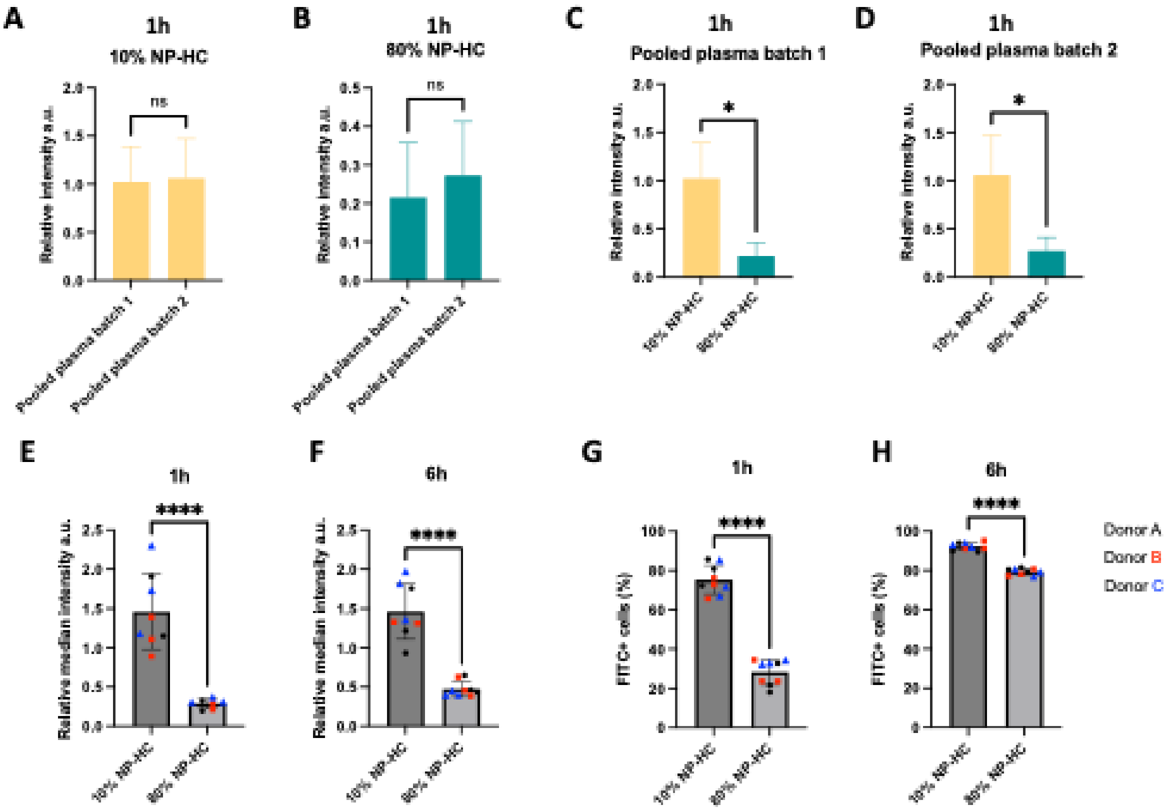

Figure SI.6: *Cellular uptake of NP- HC complexes across plasma batches and donors. Uptake of 10% (yellow) and 80% (green) NP-HC complexes, respectively, after 1 hour of incubation, across two independent plasma batches (batch 1, collected in 2017 and batch 2, collected in 2020) (A and B, respectively). Comparison of 10% and 80% NP HC uptake within the same plasma batch to assess condition-specific differences after 1 hour (C and D). Uptake profiles from three individual donor samples at 1 hour and 6 hours, measured by Relative Median Fluorescence Intensity (E and F) and percentage of FITC positive cells (G and H). Data show consistent trends across donors and timepoints. Statistical analysis was performed using paired t-test; ns= not significant, *p < 0.05, ****p < 0.0001.*

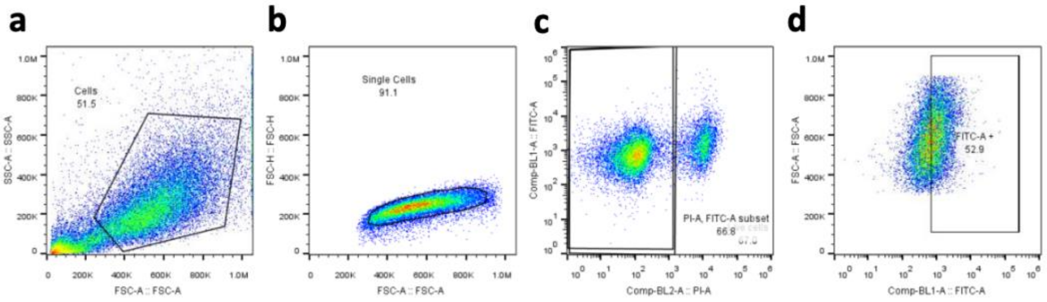

Figure SI.7: *Figure SI.7. Flow cytometry gating strategy. Cell populations were gated sequentially on forward and side scatter (a), singlets (b), live cells (c), and the final FITC positive population of interest (d).*

***Table SI 1**: Glycan structures identified in pooled plasma samples. List of N-glycans identified in the pooled plasma glycan profile based on retention times and structure assignments using Ludger reference standards.*

Peak number	Glycan structure					
	#1	#2	#3	#4	#5	#6
1	FA1					
2	A2					
3	A2	A1[6]G1	A2B	FA2		
4	M2A1G1S1					
5	M5	A2[6]G1				
6	FA2	FA2B	M5			
7	FA1[6]G1	A2[6]G1				
8	A2[6]G1					
9	A2[6]G1	A2[6]BG1				
10	FA1[6]G1	A2[6]BG1	FA2[6]G1	M4A1G1		
11	FA2[6]G1					
12	FA2[6]G1	FA2[6]BG1	M6 D1 OR D2	A1[3]G1S[3]1		
13	FA2[6]BG1	M6 D1 OR D2	A1[3]G1S[3]1			
14	A2G2					
15	A2BG2	A2[3]G1S[3]1	FA1G1S[3]1			
16	A2[3]G1S[3]1	A2BG1S1	FA1G1S[3]1			
17	M5A1G1	FA2G2				
18	FA2BG2					
19	A2[6]BG1S[3]1	M4A1G1S[3]1	FA2[6]G1S[3]1			
20	A2[3]G1S[3]1	FA2[6]G1S[3]1		FA2[6]BG1S[3]1		
21	M7D1					
22	FA2[6]BG1S[3]1	A2G2S[3]1				
23	A2BG2S[3]1					
24	M5A1G1S[3]1	M8 D2,D3	FA2G2S[3]1	FA2BG2S[3]1		
25	M8 D2,D3	FA2BG2S[3]1	*FA2G2S[3,3]2			
26	*A2G2S[3,3]2					
27	FA2G2S[3]1		A3G3S[3]1			
28	M5BG1S1					
29	*FA2G2S[3,3]2	*A2G2S[3,3]2				
30	*A2G2S[3,3]2	A3G3S[3]1	FA3G3S[3]1			
31	A2BG2S[3,3]2					
32	A2G2S[3]1	*FA2G2S[3,3]2	A3G3S[3]1	FA3G3S[3]1		
33	*FA2G2S[3,3]2					
34	FA3G3S[3]1	FA2BG2S[3,3]2				
35	A3G3S[3]1	FA3G3S[3]1	A3G3S[3,3]2			
36	A2G2S[3]1	A3G3S[3,3]2				
37	A3G3S[3,3]2	A4G4S[3]1	FA3G3S[3,3]2			
38	A2G2S[3]1	A3G3S[3,3]2	FA3G3S[3,3]2			
39	A2G2S[3]1	FA3G3S[3]1	A3G3S[3,3]2	A4G4S[3]1	FA3G3S[3,3]2	*A3G3S[3,3,3]
40	FA3G3S[3,3]2					
41	FA3G3S[3,3]2	*A3G3S[3,3,3]3				
42	A3G3S[3,3]2	FA3G3S[3,3]2	*A3G3S[3,3,3]3			

Table SI 2: *Glycan profiles of 10% NP HC and 80% NP HC. List of N-glycans identified for the 10% and 80% NP based on retention times and structure assignments using the full plasma reference standard.*

Human plasma HC peaks		Structures					
**10% NP-HC**	**80% NP-HC**	**#1**	**#2**	**#3**	**#4**	**#5**	**#6**
**3**	**3**	A2	A1[6]G1	A2BFA2			
**10**		FA1[6]G1	A2[6]BG1	FA2[6]G1	M4A1G1		
**11**		FA2[6]G1					
**12**		FA2[6]G1	FA2[6]BG1	M6 D1 OR D2	A1[3]G1S[3]1		
**14**		A2G2					
**16**		A2[3]G1S[3]1	A2BG1S1	FA1G1S[3]1			
**17**		M5A1G1	FA2G2				
**18**	**18**	FA2BG2					
**22**	**22**	FA2[6]BG1S[3]1		A2G2S[3]1			
**24**	**24**	M5A1G1S[3]1	M8 D2,D3	FA2G2S[3]1	FA2BG2S[3]1		
**25**		M8 D2,D3	FA2BG2S[3]1	*FA2G2S[3,3]2			
**26**	**26**	*A2G2S[3,3]2					
**30**	**30**	*A2G2S[3,3]2		A3G3S[3]1	FA3G3S[3]1		
**33**	**33**	*FA2G2S[3,3]2					
**35**	**35**	A3G3S[3]1	FA3G3S[3]1	A3G3S[3,3]2			
**36**		A2G2S[3]1	A3G3S[3,3]2				
**38**	**38**	A2G2S[3]1	A3G3S[3,3]2	FA3G3S[3,3]2			
**39**	**39**	A2G2S[3]1	FA3G3S[3]1	A3G3S[3,3]2	A4G4S[3]1	FA3G3S[3,3]2	*A3G3S[3,3,3]3
**42**	**42**	FA3G3S[3,3]2					
